# miRNA and mRNA Profiling Links Connexin Deficiency to Deafness via Early Oxidative Damage in the Mouse *Stria Vascularis*

**DOI:** 10.3389/fcell.2020.616878

**Published:** 2021-01-25

**Authors:** Giulia Gentile, Fabiola Paciello, Veronica Zorzi, Antonio Gianmaria Spampinato, Maria Guarnaccia, Giulia Crispino, Abraham Tettey-Matey, Ferdinando Scavizzi, Marcello Raspa, Anna Rita Fetoni, Sebastiano Cavallaro, Fabio Mammano

**Affiliations:** ^1^Department of Biomedical Sciences, National Research Council (CNR) Institute for Biomedical Research and Innovation, Catania, Italy; ^2^Department of Neuroscience, Università Cattolica del Sacro Cuore, Rome, Italy; ^3^Fondazione Policlinico Universitario A. Gemelli Istituto di Ricovero e Cura a Carattere Scientifico (IRCCS), Rome, Italy; ^4^Department of Head and Neck Surgery, Università Cattolica del Sacro Cuore, Rome, Italy; ^5^Department of Biomedical Sciences, National Research Council (CNR) Institute of Biochemistry and Cell Biology, Rome, Italy; ^6^Department of Mathematics and Computer Science, University of Catania, Catania, Italy; ^7^Department of Physics and Astronomy “G. Galilei”, University of Padua, Padua, Italy

**Keywords:** connexins, molecular pathway analysis, early degeneration, systems biology, hearing loss, vascular dysfunction, post-natal development, oxidative stress

## Abstract

Pathogenic mutations in the non-syndromic hearing loss and deafness 1 (DFNB1) locus are the primary cause of monogenic inheritance for prelingual hearing loss. To unravel molecular pathways involved in etiopathology and look for early degeneration biomarkers, we used a system biology approach to analyze Cx30^−/−^ mice at an early cochlear post-natal developmental stage. These mice are a DFNB1 mouse model with severely reduced expression levels of two connexins in the inner ear, Cx30, and Cx26. Integrated analysis of miRNA and mRNA expression profiles in the cochleae of Cx30^−/−^ mice at post-natal day 5 revealed the overexpression of five miRNAs (miR-34c, miR-29b, miR-29c, miR-141, and miR-181a) linked to apoptosis, oxidative stress, and cochlear degeneration, which have Sirt1 as a common target of transcriptional and/or post-transcriptional regulation. In young adult Cx30^−/−^ mice (3 months of age), these alterations culminated with blood barrier disruption in the *Stria vascularis* (*SV*), which is known to have the highest aerobic metabolic rate of all cochlear structures and whose microvascular alterations contribute to age-related degeneration and progressive decline of auditory function. Our experimental validation of selected targets links hearing acquisition failure in Cx30^−/−^ mice, early oxidative stress, and metabolic dysregulation to the activation of the Sirt1–p53 axis. This is the first integrated analysis of miRNA and mRNA in the cochlea of the Cx30^−/−^ mouse model, providing evidence that connexin downregulation determines a miRNA-mediated response which leads to chronic exhaustion of cochlear antioxidant defense mechanisms and consequent *SV* dysfunction. Our analyses support the notion that connexin dysfunction intervenes early on during development, causing vascular damage later on in life. This study identifies also early miRNA-mediated biomarkers of hearing impairment, either inherited or age related.

## Introduction

Cx26 and Cx30 are the prevailing gap junction proteins in the duct of the developing and mature mammalian cochlea (Mammano, 2019). Pathogenic mutations in the *DFNB1* locus, which contains both genes (*GJB2/CX26* and *GJB6/CX30*) encoding these connexins, are the primary cause of monogenic inheritance for prelingual deafness. It remains unclear if their coordinated expression is due to digenic inheritance or mutations affecting *cis-*regulatory elements that in turn influence *GJB2/CX26* expression (Del Castillo and Del Castillo, 2017).

Knockout mouse models confirmed the essential role of inner ear connexins for hearing, since their absence causes profound deafness associated with apoptotic processes within the developing organ of Corti (OC) (Teubner et al., 2003). *Cx30* homozygous knockout-LacZ mice (*Gjb6*^*tm*1*Kwi*^/*Gjb6*^*tm*1*Kwi*^; MGI:2447863; EM:00323), hereafter abbreviated as *Cx30*^−/−^, are a model for humans in which large deletions in the DFNB1 locus lead to downregulation of both *GJB2/CX26* and *GJB6/CX30* and profound deafness. *Cx30*^−/−^ mice exhibit (i) severe constitutive hearing impairment with degeneration of cochlear sensory epithelium from post-natal day 18 (P18) onwards, (ii) absence of endocochlear potential (Teubner et al., 2003), and (iii) defects of the endothelial barrier in capillaries of the *stria vascularis* (*SV*) (Cohen-Salmon et al., 2007). In addition, massive downregulation of Cx26, at both mRNA and protein levels, was reported in the developing OC of *Cx30*^−/−^ mice at P5 in non-sensory cells located between outer hair cells and *SV* (Ortolano et al., 2008); both transcript and protein levels of Cx26 were similarly reduced in the cochleae of *Cx30*^−/−^ mice at P30 (Boulay et al., 2013).

The coordinated regulation of Cx26 and Cx30 expression in the cochlea apparently occurs as a result of NFκB pathway signaling, as it could be inhibited by expressing a stable form of the IκB repressor protein that prevents the activation/translocation of NFκB (Ortolano et al., 2008). The amplitude and duration of Ca^2+^ signals control differential activation of NFκB (Dolmetsch et al., 1998), and prior works linked alterations of Ca^2+^ signaling to hearing loss in transgenic mice (Schutz et al., 2010; Rodriguez et al., 2012). Other published data linked defective hearing acquisition to impairment of ATP- and IP_3_-dependent Ca^2+^ signaling in non-sensory cells of the developing cochlea (Ceriani et al., 2016), identified inner ear connexins as both targets and effectors of these signaling mechanisms, and support the notion that connexin dysfunction intervenes early on during development, calling for a timely therapeutic intervention (Crispino et al., 2011).

Recently, the emerging role of microRNAs (miRNAs) in post-transcriptional gene expression regulation has also been investigated using knockout mouse models whose miRNA deregulated profiles may suggest a potential contribution to cochlear pathogenesis (Elkan-Miller et al., 2011; Patel and Hu, 2012; Ushakov et al., 2013; Zhang et al., 2013; Mahmoodian sani et al., 2016; Mittal et al., 2019). In addition, deficiency of Cx26 in *Gjb2*/*Cx26* conditional knockout mice was linked to an impaired miRNA-mediated intercellular communication through cochlear gap junctions (Zhu et al., 2015).

Here, we explored mechanisms underlying the etiopathogenesis of DFNB1 by performing an integrated genomics analysis of miRNA and mRNA expression profiles in *Cx30*^−/−^ mice. In a prior work, transcriptomic profiles of *Cx30*^−/−^ mice (and their wild-type siblings) were obtained at P13, highlighting a significant downregulation of betaine homocysteine *S*-methyltransferase (*Bhmt*) restricted to the *SV*, followed by the consequent local increase of homocysteine level and endothelial barrier dysfunction (Cohen-Salmon et al., 2007). In this study, we extended those results by also investigating miRNA-mediated regulation at an earlier stage of cochlear development, i.e., P5, and so looking for early degeneration biomarkers.

## Materials and Methods

Electronic Laboratory Notebooks Were Not Used.

### Animals and Genotyping

Animals (*Mus musculus*) used in this study and bred at the National Research Council–Institute of Biochemistry and Cell Biology (CNR-IBBC), Infrafrontier/ESFRI–European Mouse Mutant Archive (EMMA), specific pathogen-free (SPF) barrier unit (Monterotondo Scalo, Rome, Italy), were housed in individually ventilated caging systems (Tecniplast, Gazzada, Italy) at a temperature (T) of 21 ± 2°C and relative humidity (RH) of 55 ± 15% with 50–70 air changes per hour (ACH) and under controlled (12:12 h) light–dark cycle (7 am−7 pm). Mice had *ad libitum* access to water and a standard rodent diet (Emma 23, Mucedola, Settimo Milanese, Italy). Both male and female homozygous Cx30^−/−^ [EMMA ID (EM):00323] pups at P5, as well as their wild-type P5 siblings (Cx30^+/+^), were used. The background strain of these mice was C57BL/6J.

Primer pairs for Cx30^−/−^ mice were specific for the wild-type alleles:

f: 5′-GGTACCTTCTACTAATTAGCTTGG-3′,

r: 5′-AGGTGGTACCCATTGTAGAGGAAG-3′.

To visualize the deletion, primers specific for the lacZ region (which flanks the deleted allele) were used in combination with the corresponding wild-type forward primer:

lacZ 5′-AGCGAGTAACAACCCGTCGGATTC-3′.

### Study Design

To construct the optimal experimental design and estimate the minimum number of animals necessary for the experiments (sample size of the groups), for each type of experiment and for each genetically modified and control strain, we set probability *a* = 5% = 0.05 for the type I error in the *t*-test. Then, fixing *b* = 4*a* = 20% = 0.2 to obtain a test power of 1–*b* = 80% = 0.8, we computed the number *n* of each of the two samples to be compared using the following formula:

(1) $$\begin{array}{l} n>2\left[\frac{\left(z_{\alpha /2}+z_{\beta }\right)\cdot \sigma^{2}}{\Delta }\right] \end{array}$$

with *z*_*a*__/2_ = 1.96 and *z*_*b*_ = 0.842. Based on experiments of the same type carried out in prior work, we quantified the variability of the data (variance, σ^2^) and established the minimum difference, Δ = |μ_1_-μ_2_|, between averages that had a biological significance. To minimize subjective bias, sample identity (e.g., genotypes) was randomized by associating an identification number to each sample before processing. One sample was excluded from the analysis after microarray quality control in miRNA expression profiling.

### RNA Extraction and Evaluation

To study gene expression regulation by miRNAs during cochlear development in Cx30^−/−^ mice, we performed an integrated functional genomics analysis (for data analysis flow chart, see Supplementary Figure 1). We used both cochleae of *n* = 4 mice at P5 for each of the two genotypes and extracted total RNA, including miRNAs using the Qiagen miRNeasy Mini Kit (Qiagen, Hilden, Germany). The quality of RNA was evaluated with Agilent 2100 Bioanalyzer, using the Small RNA assay for miRNAs and the RNA 6000 Nano assay for mRNAs. All of the eight samples thus obtained passed the quality control and were processed for both types of profiling experiments as described below.

### miRNA Expression Profiling

miRNAs were labeled and purified starting from 100 ng of total RNA of each sample and then hybridized on Mouse miRNA Microarrays v.21.0, 8 × 60 K (Agilent Technologies), according to the miRNA Complete Labeling and Hyb Kit Protocol (Version 3.1.1, August 2015). Microarrays were scanned at 3-μm resolution using a SureScan Microarray Scanner System (Agilent Technologies), and the Feature Extraction Software v. 11.5.1.1 (Agilent Technologies) was used for acquisition, data extraction, and quality control analysis. After the evaluation of quality control parameters for each scanned microarray image, the raw data of three replicates for wild-type and four replicates for knockout experimental conditions were analyzed using the Gene-Spring GX v.14.5 software (Agilent Technologies), by which fluorescence signal values were set at a threshold of 1, log2 transformed, and normalized using the 90th percentile shift method. The resulting data were baselined to the median of all samples and quality-filtered on flags to include any probe detected in 100% of biological replicates in at least one out of the two tested experimental conditions. Significantly deregulated miRNAs were identified using a moderate paired *t*-test and Benjamini–Hochberg multiple testing correction. MicroRNA profiling data were submitted to GEO (GSE151368).

### Whole-Genome Transcription Profiling

Messenger RNA was labeled and purified starting from 100 ng of total RNA of each sample and then hybridized on SurePrint G3 Mouse Gene Expression v2 8 × 60 K Microarrays (Agilent Technologies), according to the One-Color Microarray-Based Gene Expression Analysis—Low Input Quick Amp Labeling kit protocol (Version 6.9.1, December 2015). Microarrays were scanned at 3-μm resolution using a SureScan Microarray Scanner System (Agilent Technologies), and the Feature Extraction Software v. 11.5.1.1 (Agilent Technologies) was used for acquisition, data extraction, and quality control analysis. The raw data of all samples were analyzed using the Gene-Spring GX v.14.5 software (Agilent Technologies). Fluorescence signal values were set at a threshold of 1, log2 transformed, normalized to the 75th percentile, baselined to the median of all samples, and quality-filtered on flags to include any probe detected in 100% of biological replicates in at least one out of the two tested experimental conditions. Differentially expressed genes (DEGs) were identified by a moderate *t*-test and Westfall–Young multiple testing correction. Data were submitted to GEO (GSE151367).

To perform gene set-focused expression analysis, we selected two gene lists from the Gene Ontology (GO) Resource knowledgebase (http://geneontology.org/) and its AmiGO tool (http://amigo.geneontology.org/amigo) using “glutathione” and “homocysteine” as keywords and filtering for *M. musculus*. The two selected gene lists made up of glutathione-related genes (n.86) and homocysteine-related (n.17) genes are, respectively, available in Supplementary Tables 1, 2. To identify DEGs between knockout and wild-type animals in each gene set-focused list, a moderate *t*-test was applied without correction.

### Functional Annotation and Enrichment

To functionally annotate DEGs and their products, we used the DAVID Bioinformatics Resources v. 6.8 public database (https://david.ncifcrf.gov/), together with the MetaCore software from Clarivate Analytics (https://portal.genego.com/); Mouse Genome Database (http://www.informatics.jax.org/); and the UniProt knowledgebase (http://www.uniprot.org).

### Integrated Analysis of miRNA and mRNA Expression

To identify miRNA targets, we used the list of significant differentially expressed miRNAs as input queries for the DIANA-TarBase v7.0 tool (http://www.microrna.gr/tarbase), which contains high-quality manually curated experimentally validated miRNA–gene interactions inferred from published data, using the species *M. musculus* and 30 validation methods as filters. To annotate deregulated miRNAs with no validated gene targets in the DIANA-TarBase, we used three tools for miRNA target prediction, miRWalk v2.0 (http://zmf.umm.uni-heidelberg.de/apps/zmf/mirwalk2/), miRDB (http://www.mirdb.org/), and RNA22 v2.0 (https://cm.jefferson.edu/rna22/), selecting target genes if present in two out of three of them (Supplementary Table 3). The list of validated target genes for each miRNA was used as input for the target network analysis described below.

### Network Analysis

To better clarify the interactions between gene targets of deregulated miRNAs at protein level, an extended protein–protein interaction (PPI) network was built using the STRING database v.10.0, visualized with the Cytoscape v.3.4.0 software, and analyzed through its Network Analyzer plug-in. A network was built using 2,914 miRNA target genes as seed molecules (the entire workflow of analysis, setting, and filter values are summarized in Supplementary Figure 2). A network topology analysis was also performed on the base of topological parameters to identify hub nodes or proteins having a higher degree of connectivity reflecting their biological relevance. The final PPI network was visualized on the base of node degree and edge betweenness parameters. The relative importance of the network proteins was determined based on the node centrality measure and setting the topological parameter “node degree” to ≥10. Likewise, values of edge betweenness (≥50) were mapped with the edge size: high values of this parameter correspond to a large edge size, where edge indicates interactions.

### qPCR Quantitative Analysis of Cx26, Cx30, and p53 Transcript Levels

RNA was extracted from whole cochleae freshly isolated from Cx30^+/+^ and Cx30^−/−^ mice at P5 using an RNeasy kit (Cat. No. 74104, Qiagen, Milan, Italy). cDNA was obtained by reverse transcription of mRNA with Oligo(dT)12–18 (Cat. No. 18418012, Thermo Fisher Scientific, Milan, Italy) and OmniScript Reverse Transcriptase (Cat. No. 205111, Qiagen) for 1 h at 37°C. qPCR was performed on cDNA to amplify Cx26, Cx30, and p53 and normalized to GAPDH expression (Pfaffl, 2001). Amplification was carried out using SYBR Green (Cat. No. 4367659, Applied Biosystems) on the ABI 7700 sequence detection system equipped with the ABI Prism 7700 SDS software using the following amplification cycles:

50°C, 2 min;
95°C, 10 min;
95°C, 15 sec; and
60°C, 1 min (40 cycles).
Primers used are listed as follows:
Cx26f: 5′-TCACAGAGCTGTGCTATTTG-3′
Cx26r: 5′-ACTGGTCTTTTGGACTTTCC-3′
Cx30f: 5′-GGCCGAGTTGTGTTACCTGCT-3′
Cx30r: 5′-TCTCTTTCAGGGCATGGTTGG-3′
p53f: 5′-GTATTTCACCCTCAAGATCC-3′
p53r: 5′-TGGGCATCCTTTAACTCTA-3′
GAPDHf: 5′-ATGTGTCCGTCGTGGATCTGAC-3′
GAPDHr: 5′-AGACAACCTGGTCCTCAGTGTAG-3′.

### Immunohistochemistry and Confocal Imaging

Animals were terminally anesthetized (ketamine, 70 mg/g for males and 100 mg/g for females, and medetomidine 1 mg/g), their cochleae were quickly removed, and samples were fixed with 4% paraformaldehyde in phosphate-buffered saline (PBS) at 4°C and pH 7.5. For all immunofluorescence analyses, control experiments were performed by omitting the primary antibody during the processing of tissues, randomly selected across experimental groups. Staining was absent in these control cochlear samples, indicating neither autofluorescence nor lack of antibody specificity (data not shown). Tissues from all groups were always processed together to limit variability related to antibody penetration, incubation time, post-sectioning age, and condition of tissue.

### Cx26 and Cx30 Immunofluorescence Analysis

To evaluate Cx30 and Cx26 expression in cochlear structures, cochleae from Cx30^+/+^ and Cx30^−/−^ mice were decalcified for 3 days in EDTA (0.3 M). Specimens were included in 3% agarose dissolved in PBS and cut into 100-μm thickness steps using a vibratome (VT 1000 S, Leica). Tissue slices were permeabilized with 0.1% Triton X-100, dissolved in 2% bovine serum albumin solution. Cx26 and Cx30 were immunolabeled by overnight incubation at 4°C with mouse monoclonal selective antibodies (Cx26, 10 μg/ml, Thermo Fisher, Cat. No. 335800; Cx30, 10 μg/ml, Thermo Fisher, Cat. No. MA5-35021) followed by incubation with a goat anti-mouse IgG secondary antibody (10 μg/ml, Alexa Fluor® 488, Thermo Fisher, Cat. No. A11029), applied at room temperature (22–25°C). F-actin was stained by incubation with Alexa Fluor 568 phalloidin (1 U/ml, Thermo Fisher, Cat. No. A12380), and nuclei were stained with 4′,6-diamidino-2-phenylindole (DAPI, Thermo Fisher, Cat. No. D1306) (1:200).

### DHE Staining on Cochlear Tissue Cryosections

Cochleae of Cx30^+/+^ and Cx30^−/−^ terminally anesthetized mice were quickly removed and fixed with 4% paraformaldehyde in PBS at 4°C. Next, cochleae were decalcified in 10% EDTA (changed daily), incubated for 48 h in sucrose (30%), embedded in OCT and cryosectioned (6 μm). To evaluate the superoxide amount, cochlear slices were incubated with 1 mM DHE (Cat. No. D23107, Thermo Fisher) in PBS for 30 min at 37°C, embedded in antifade medium, and sealed with coverslips. DHE was imaged using an ultrafast tunable mode-locked titanium: sapphire laser (Chameleon; Coherent, 792 nm, 140 fs, 80 MHz) coupled to a multiphoton microscope (Nikon) equipped with a 20 × Plan Apo objective (0.75 NA, Nikon).

### p53 and Sirt1 Immunofluorescence

Cochlear cryosections (6 μm) were first treated with a blocking solution (1% BSA, 0.5% Triton X-100, and 10% normal goat serum in PBS 0.1 M) and then incubated overnight at 4°C with a solution containing primary antibodies against p53 (Cat. No. #2524, Cell Signaling Tech, Boston, MA USA, diluted 1:100 in PBS) and Sirt1 (Cat. No. #9475, Cell Signaling Tech, diluted 1:100 in PBS). Next, specimens were incubated at room temperature for 2 h in labeled-conjugated donkey anti-rabbit and/or anti-mouse secondary antibody (Alexa Fluor 488 or 546, IgG, Thermo Fisher, diluted 1:400 in PBS) and counterstained with DAPI (Cat. No. D1306, Thermo Fisher; 1:500). Images were obtained with the confocal laser scanning system (Nikon Ti-E, Confocal Head A1 MP, Japan) equipped with an Ar/ArKr laser (for 488-nm excitation), an HeNe laser (for 543-nm excitation), and a 20 × Plan Apo objective (0.75 NA, Nikon). DAPI was imaged by two-photon excitation (740 nm, <140 fs, 90 MHz as detailed above).

### Extravasation Assay

Cx30^+/+^ and Cx30^−/−^ mice at 3 months of age (3m) were anesthetized by intraperitoneal injections of ketamine (35 mg/kg) and medetomidine (1 mg/kg). Next, 30 μl of dye-containing solution (Texas Red™ dextran 70,000 MV, Invitrogen, Cat. No. 1987295, dissolved in PBS at a concentration of 2.5 mg/ml) was injected via the tail vein. After 3 min, injected animals were euthanized by cervical dislocation, cochleae were dissected in ice-cold PBS, and the spiral ligament and *SV* were microdissected from the rest of the cochleae. *SV* strips were detached from the spiral ligament and mounted onto glass slides with a mounting medium (FluorSave™ Reagent, Cat. No. 345789-20M, Merck). Images were obtained with a confocal laser scanning system mentioned above equipped with a 20 × dry objective (20X PL Fluotar 0.5, NA, Leica). The fluorescent intensity of each area of interest was quantified with ImageJ (version 2.0.0-rc-69/1.53c), and statistics were computed using MATLAB R2019b on *n* = 3 mice for each genotype.

### Western Immunoblots

Total proteins were extracted from cochleae of Cx30^+/+^ and Cx30^−/−^ animals (*n* = 8 animals per group). Cochleae were dissected, collected on ice, stored at −80°C, and homogenized by using ice-cold RIPA buffer [Pierce: 50 mM Tris, 150 mM NaCl, 1 mM EDTA, 1% DOC, 1% Triton X-100, 0.1% SDS, 1 × protease, phosphatase-1, and phosphatase-2 inhibitor cocktails (Merck)]. The lysate was sonicated three times at 10 Hz (Hielscher, Ultrasound Technology UP50H/UP100H), centrifuged (13,000 rpm, 15 min, 4°C), and a 5-μl aliquot of the supernatant was assayed to determine the protein concentration (microBCA kit, Pierce). Reducing sample buffer was added to the supernatant, and samples were heated to 95°C for 5 min. Protein lysates (70 μg) were loaded onto Tris-glycine polyacrylamide gels for electrophoretic separation. Colorburst™ electrophoresis markers (Bio-Rad or Amersham) were used as molecular mass standards. Proteins were then transferred onto nitrocellulose membranes at 100 V for 2 h at 4°C in transfer buffer containing 25 mM Tris, 192 mM glycine, 0.1% SDS, and 20% methanol. Membranes were incubated for 1 h with blocking buffer (5% skim milk in TBST) and then incubated overnight at 4°C with the following primary antibodies: anti-Cx26 (mouse monoclonal, Cat. No. 335800, Thermo Fisher Scientific); anti-Cx30 (mouse monoclonal, Cat. No. MA5-35021, Thermo Fisher Scientific); anti-p53 (mouse monoclonal, Cat. No. #2524, Cell Signaling Tech); and anti-Sirt1 (rabbit polyclonal, Cat. No. 07-131, Merck Millipore). After three 10-min rinses in TBST, membranes were incubated for 1 h at RT horseradish peroxidase (HRP)-conjugated mouse or rabbit secondary antibodies (Cat. No. #7076 and Cat. No. #7074, respectively, Cell Signaling, 1:2,500). Equal protein loading among individual lanes was confirmed by reprobing the membranes with an anti-GAPDH (1:10,000, Cat. No. ab8245, Abcam) or anti-tubulin mouse monoclonal antibody (1:10,000, Cat. No. T6074, Sigma). Membranes were then washed and bands visualized with an enhanced chemiluminescence detection kit (Cat. No. K-12045-D50, Advansta). Protein expression was evaluated and documented by using UVITEC (Cambridge Alliance).

### Statistical Analysis

For normally distributed data, statistical comparisons of means data were made by Student's two-tailed *t*-test using a worksheet (Microsoft Office Excel 2017, Version 1.30), whereas ANOVA and *post*-*hoc* comparison by Tukey's test were used to analyze the differences among group means using Statistica (version 6.0, StatSoft Inc.). The same software was also used to perform the Mann–Whitney *U*-test on data that did not require the assumption of normal distribution. Mean values are quoted ± standard error of the mean (s.e.m.) where *p* < 0.05 indicate statistical significance.

### Study Approval

All experimental protocols involving the use of animals (*M. musculus*) were approved by the Ethical Committee of Padua University (Comitato Etico di Ateneo per la Sperimentazione Animale, C.E.A.S.A., Project no. 58/2013, protocol no. 104230) and the Italian Ministry of Health (DGSAF 0001276-P-19/01/2016 and 68/2016-PR). Experimental procedures were also agreed upon, reviewed, and approved by local animal welfare oversight bodies and were performed with the approval and direct supervision of the CNR-IBBC/Infrafrontier—Animal Welfare and Ethical Review Body (AWERB), in accordance with general guidelines regarding animal experimentation, approved by the Italian Ministry of Health, in compliance with the Legislative Decree 26/2014, transposing the 2010/63/EU Directive on protection of animals used in research. This work was also conducted based on recommendations from both ARRIVE and PREPARE guidelines.

## Results

### Identification of Early Degeneration Biomarkers and Deregulated Molecular Pathways at P5

#### Deregulation of miRNAs and the PPI Network of Their Targets

**Figure 1:**
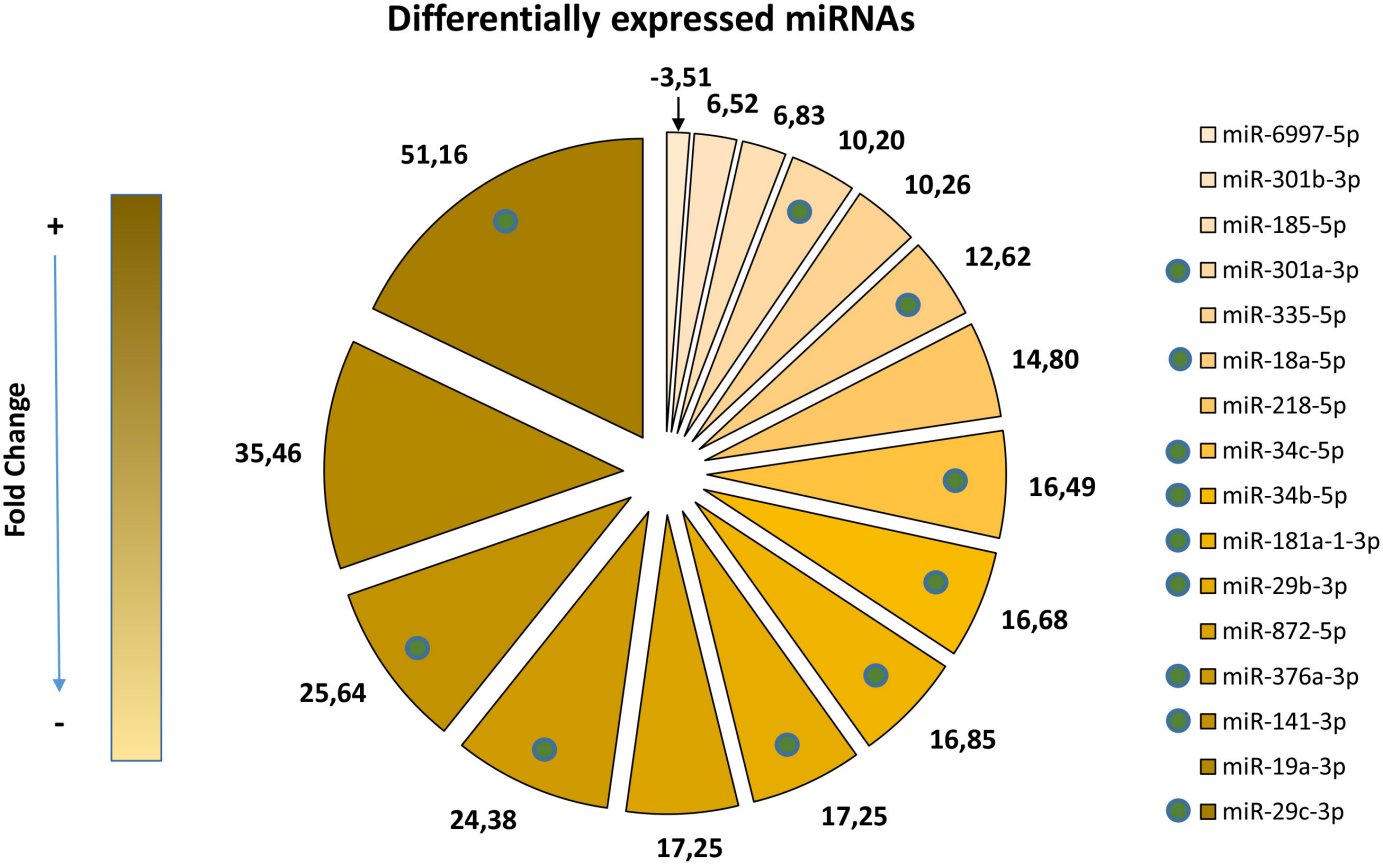
Differentially expressed miRNAs. The pie chart shows significant changes in expression of 16 miRNAs, colored from light to dark gold according to their fold change expression value in *Cx30*^−/−^ mice vs. controls with normal *Gjb6* alleles (*Cx30*^+/+^). The fold change values are shown in clockwise order starting from the smallest one, indicating with an arrow the corresponding pie chart portion. Green tags highlight miRNAs already known as linked to apoptosis or deregulated in the degeneration of the cochlea during aging. Two of these upregulated miRNAs were already found as deregulated in sensorineural diseases of the ear, i.e., miR-18a and miR-376a, in mouse inner ear spatial expression patterns at P0, as previously reviewed (Ushakov et al., 2013; Mahmoodian sani et al., 2016). Moreover, the upregulation of miR-29b was detected in mouse vestibular sensory epithelium at P2 (Elkan-Miller et al., 2011) and in the degeneration of the mouse OC during aging (at 3m, 9m, and 16m compared to P21) (Zhang et al., 2013). Four other miRNAs, miR-29c, miR-34c, miR-141, miR-181a, and miR-301a, were previously described as dysregulated in mouse OC with age-related hearing loss (Zhang et al., 2013). In particular, miR-29b/c, miR-34a/c, and miR-181a are reported as pro-apoptotically deregulated in deafness due to aging (Khanna et al., 2011; Zhang et al., 2013).

By comparing miRNA expression profiles at P5 across genotypes (*Cx30*^−/−^ vs. *Cx30*^+/+^, see Materials and Methods and Supplementary Figures 1, 2), we identified a total of 16 deregulated miRNAs. As illustrated in Figure 1 and reported in Table 1, 9 out of 16 overexpressed miRNAs (miR-18a-5p, miR-29b-3p, miR-29c-3p, miR-34a-5p, miR-34b-5p, miR-141-3p, miR-181a-1-3p, miR-301a-3p, miR-376a-3p) have been previously linked to apoptosis, oxidative stress, and degeneration of the cochlea during aging. Thus, our results suggest that connexin downregulation and/or dysfunction determines a miRNA-mediated response during early post-natal development, which may influence apoptosis, oxidative stress, and degeneration processes and which can be detected already at P5. This is much earlier than the time of cochlear sensory epithelium degeneration, which occurs from P18 onwards in this mouse model (Teubner et al., 2003).

**Table 1:** Deregulated miRNAs.

**Up-regulated miRNAs in Cx30^**KO**^ vs. Cx30^**WT**^**	**miRNA detection**	**miRNAs:*****Sirt1*** **known influence**	
**34c**	Degeneration of the OC during ARHL (Zhang et al., 2013); kanamycin-induced apoptosis of inner ear cells (Yu et al., 2010); ketamine-induced neurotoxicity in neonatal mice hippocampus (Cao et al., 2015); development, cancerous, and non-cancerous diseases (Rokavec et al., 2014).	Cognitive decline in mouse hippocampus (Zovoilis et al., 2011); neuropathic pain in rat models with chronic constriction injury of the sciatic nerve (Mo et al., 2020).	Post-transcriptional inhibition (Zovoilis et al., 2011); transcriptional repression (Mo et al., 2020).
**29b**	Degeneration of the OC during ARHL (Zhang et al., 2013); mammalian inner ear (Elkan-Miller et al., 2011); glucocorticoid-induced apoptosis in human plasmacytoid dendritic cells (Hong et al., 2013).	Cochlear hair cell apoptosis in ARHL (Xue et al., 2016); oxidative stress in ovarian cancer (Hou et al., 2017); mouse embryonic stem cells in response to ROS (Xu et al., 2014).	Transcriptional repression (Xu et al., 2014; Xue et al., 2016; Hou et al., 2017).
**29c**	Degeneration of the OC during ARHL (Zhang et al., 2013); glucocorticoid-induced apoptosis in human plasmacytoid dendritic cells (Hong et al., 2013).	Tumor suppressor in hepatocellular carcinoma (Bae et al., 2014).	Post-transcriptional inhibition (Bae et al., 2014).
**141**	Degeneration of the OC during ARHL (Zhang et al., 2013); oxidative stress in auditory cells (Wang et al., 2010).	Autophagic response in hepatocytes (Yang et al., 2017); tumor suppressor in colorectal carcinoma cells (Sun et al., 2019).	Transcriptional repression (Yang et al., 2017; Sun et al., 2019).
**181a**	Degeneration of the OC during ARHL (Zhang et al., 2013); oxidative stress in auditory cells (Wang et al., 2010); Forskolin-treated basilar papillae (Frucht et al., 2010); hair cell regeneration in the avian auditory epithelium (Frucht et al., 2011); apoptosis and survival in the brain of calorie-restricted mice (Khanna et al., 2011).	Hepatic insulin signaling and glucose homeostasis (Zhou et al., 2012).	Post-transcriptional inhibition (Zhou et al., 2012).

*The table lists deregulated miRNAs having Sirt1 as a common gene target and the model and pathologies in which their dysregulation has been detected. The mode of action of each miRNA on Sirt1 is also listed*.

The miR-29b/c controls glucocorticoid-induced apoptosis in human plasmacytoid dendritic cells (Hong et al., 2013). In mice, overexpression of miR-34a/c has been involved in drug-induced hippocampal neurodegeneration (Cao et al., 2015), whereas overexpression of miR-34a and miR-34c has been implicated in drug-induced hearing loss (i.e., aminoglycoside-mediated ototoxicity) linked to dose-dependent apoptosis of inner ear cells (Yu et al., 2010). Finally, miR-141 and miR-181a were also found differentially expressed in reactive oxygen species (ROS)-damaged auditory cells (Wang et al., 2010).

To investigate the role of deregulated miRNAs, we searched the databases for their known validated targets (Supplementary Table 3). Five of these miRNAs (miR-34c, miR-29b, miR-29c, miR-141, and miR-181a) were previously linked to OC degeneration during age-related hearing loss (ARHL, also known as presbycusis) (Zhang et al., 2013) and share *Sirt1* as a common silencing gene target, which they are known to inhibit transcriptionally and/or post-transcriptionally in both humans and mice (Table 1). *Sirt1* encodes for the NAD^+^-dependent deacetylase sirtuin 1 (Sirt1), a longevity modulator that exerts its deacetylation activity on several targets related to oxidative stress, inflammation, and apoptosis (Choi and Kemper, 2013). Like other sirtuins, it regulates antioxidant defense mechanisms involving antioxidant response elements (Singh et al., 2018). Sirt1 expression decreases with aging, whereas its increment has an antiaging role through multiple targets, including NFκB, p53, and PGC1α. Therefore, Sirt1 activation is thought to prolong life span and ameliorate age-related conditions (Chen et al., 2020b).

Two out of three of the miRNAs deregulated in our model (miRNA-34b-5p and miRNA-34c-5p) belong to the miRNA-34 family. Both are located in the same locus and coordinately expressed as a miRNA cluster in both humans and mice (Corney et al., 2007; He et al., 2007). Prior work implicated the miR-34a/Sirt1/p53 signaling pathway in cochlear cell apoptosis in an ARHL mouse model (Xiong et al., 2015). Although we did not detect differential expression of the miR-34a transcript in our model, the seed sequence of miR-34a and miR-34c is identical, suggesting that they can have the same targets (Rokavec et al., 2014). In fact, as shown in Table 1, *Sirt1* is a target also of miR-34c.

**Figure 2:**
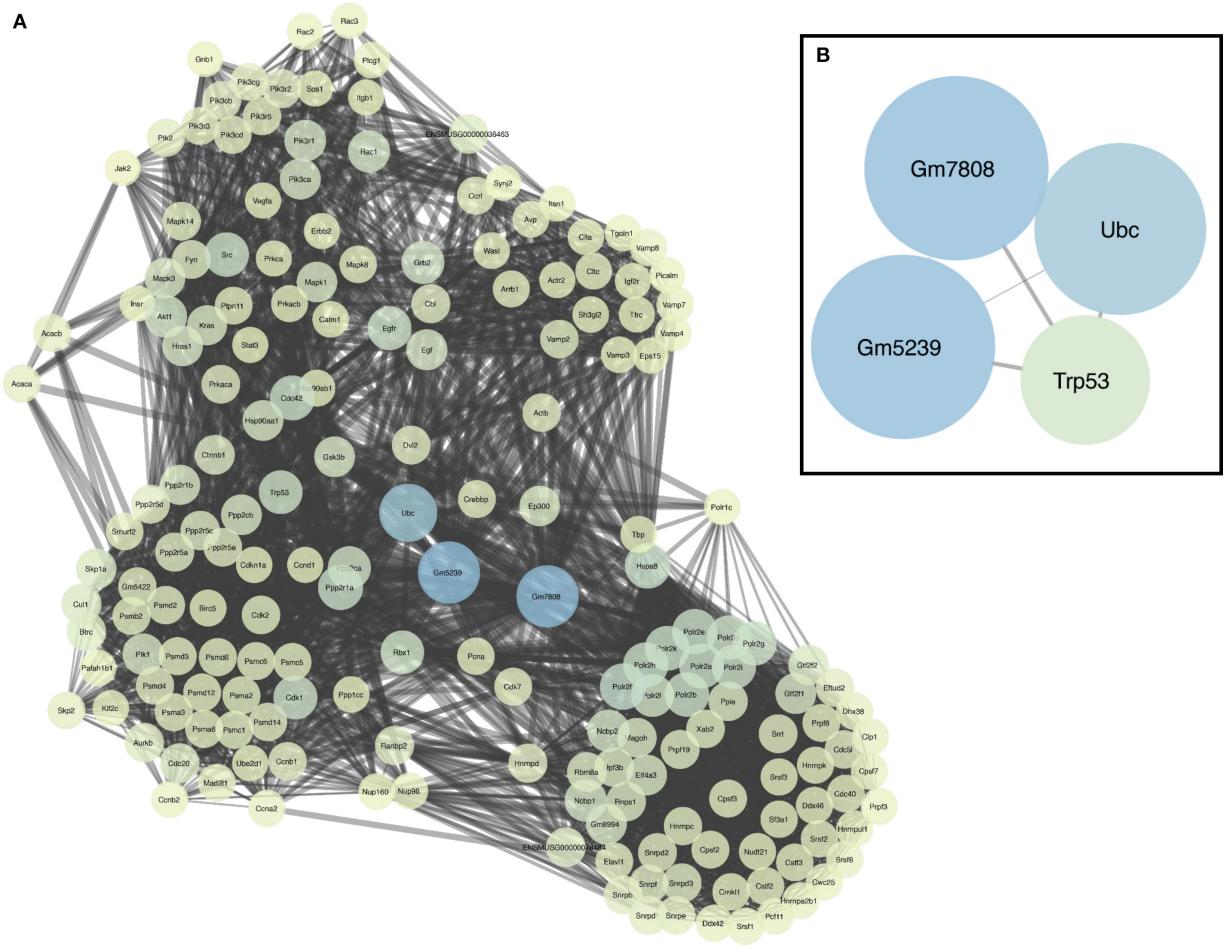
PPI network. **(A)** Extended PPI network obtained using the miRNA target genes as input data (seeds). **(B)** A focus on network hubs among which is the transcription factor p53.

We also generated a PPI network downstream of miRNA deregulation, using network analysis of miRNA targets (Figure 2A; the entire lists of network nodes and their hubs are available in Supplementary Tables 4, 5, respectively). In this scheme, the transcription factor p53 occupies a key position as a network hub downstream of miRNA deregulation (Figure 2B).

Taken together, these data suggested the involvement of a Sirt1–p53 axis downstream miRNA deregulation. Data regarding gene and protein expression of Sirt1 and p53 are provided in the sections below, related to the experimental validation of selected targets.

#### Transcriptional Profiling

We singled out 81 DEGs, among which 57 encode 56 proteins (Supplementary Table 6), and 15 correspond to non-coding RNAs (Supplementary Table 7). The co-regulated expression of *Cx30* and *Cx26*, previously described at the mRNA level (Ortolano et al., 2008), emerged also in these results, confirming that both connexins are downregulated in *Cx30*^−/−^ mice. *Cx30*^−/−^ and *Cx30*^+/+^ mice expressed similar steady-state levels of *Sirt1* and *Tp53* mRNA, suggesting that deregulation of the Sirt1–p53 axis could be controlled post-transcriptionally by miRNAs, if present.

Our gene expression profiling also identified four downregulated transcripts, *Ang, Ang4*, and *Ang6*, which encode for three proteins belonging to the RNase A superfamily (Cho et al., 2005) (Supplementary Table 6). Of note, angiogenin (ANG) is a blood-vessel-inducing protein also expressed in vascular endothelial cells (Shimoyama, 2011). In addition, Ang4 is known to have an angiogenic role (Crabtree et al., 2007). Sirt1-dependent angiogenesis, where Sirt1 is a downstream mediator of angiogenic signals regulating vascular remodeling, was reported in mouse muscle vascularization. In particular, the loss of endothelial Sirt1 resulted in an early decline of skeletal muscle vascular density, while its overexpression had a protective effect (Das et al., 2018). Moreover, we report the overexpression of *Tnfrsf10b* (Supplementary Table 6), the tumor necrosis factor receptor superfamily member 10b gene encoding for a death receptor that is a known target of p53 (Speidel, 2010).

An increment of homocysteine caused by downregulation of *Bhmt*, a gene encoding betaine-homocysteine *S*-methyltransferase 1, was previously reported in P13 *Cx30*^−/−^ mice and correlated to endothelial dysfunction in the *SV* (Cohen-Salmon et al., 2007). Here, we report downregulation of *Bhmt* already at P5 (Supplementary Table 6). This zinc metalloenzyme belongs to the trans-sulfuration pathway and catalyzes the subsequent conversion of methionine to homocysteine and cysteine, upstream of glutathione synthesis by glutathione synthetase (Gss) (Garrow, 1996). Importantly, we recently found deregulation of glutathione metabolism in *Cx26*^−/−^ mice, in which reduced release of glutathione from connexin hemichannels has been also attributed to downregulation of *Gss* (Fetoni et al., 2018), a glutathione-related gene.

In light of these results, we performed a gene set-focused expression analysis on glutathione and homocysteine-related genes (Supplementary Tables 1, 2). We detected the downregulation of *Gss* and the dysregulation of two other homocysteine-related genes, i.e., overexpression of C-1-tetrahydrofolate synthase (*Mthfd1*) and downregulation of *Bhmt*. The C-1-tetrahydrofolate synthase is a trifunctional protein involved in the tetrahydrofolate interconversion pathway, which interacts with the trans-sulfuration pathway at the homocysteine level (Ducker and Rabinowitz, 2017; Sbodio et al., 2019). Taken together, the results of this analysis support the involvement of homocysteine metabolism dysregulation in hearing loss.

#### MiR-34c-5p and Cx26

To identify possible transcriptional regulation between deregulated miRNAs and mRNAs, we compared the two lists of differentially expressed entities. This analysis yielded a matched list of differentially expressed miRNAs and their differentially expressed targets (Supplementary Table 8), among which only the downregulation of *Cx26* mediated by miR-34c-5p showed inverse deregulation between the overexpressed miRNA compared to its downregulated target gene. This direct regulation is reported in the DIANA-TarBase v7.0 tool, as inferred by crosslinking immunoprecipitation followed by RNA-seq in mouse regenerating liver (Schug et al., 2013). This miRNA–mRNA interaction was also predicted by the miRDB and RNA22 v2.0 databases.

The integration of miRNA profiling and transcriptional target analysis with gene expression profiling (taking into account pairs of experimentally validated miRNA/mRNA with an inverse correlation between their expression levels) suggested that transcriptional repression of *Cx26* could be modulated by miR-34c-5p action. In addition, other downregulated genes identified by our transcriptional profiling (Supplementary Table 6) have sequences complementary to miR-34c/34b, including *Trpa1, Trpm1, Ang, Ang4*, and *Ang6*, which are predicted interactors of miR-34c/miR-34b in the RNA22 v2.0 database.

### Oxidative Stress and Vascular Dysfunction

#### Immunofluorescence and Western Immunoblotting Analyses at P5

**Figure 3:**
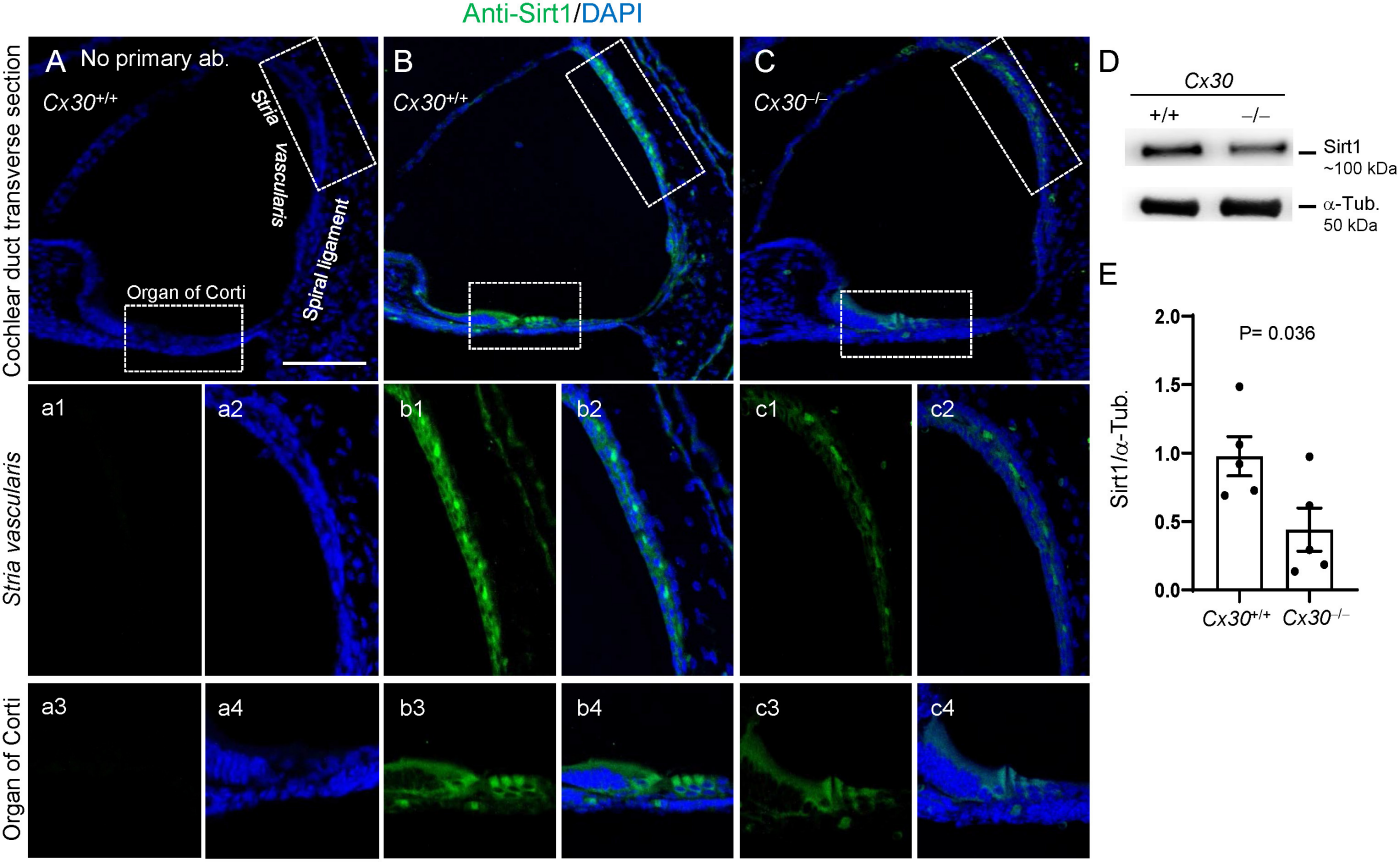
Sirt1 expression in *Cx30*^+/+^ and *Cx30*^−/−^ cochleae at P5. **(A–C)** Representative images of immunofluorescence analysis with Sirt1-selective antibodies in the cochlear sensory epithelium and lateral wall of *Cx30*^+/+^ and *Cx30*^−/−^ **(B,C)** specimens. Images of negative control performed by omitting primary Sirt1 antibody are shown in **(A)**. **(a1–c4)** Higher magnification images of the OC and *SV* (dotted box in **A–C**) showing separately Sirt1 fluorescence **(a1,b1,c1,a3,b3,c3)** and merged Sirt1/DAPI nuclei staining **(a2,b2,c2,a4,b4,c4)**. Scale bar: 100 μm. **(D)** Representative western blot immunoreactive bands quantifying the expression of Sirt1 in *Cx30*^+/+^ and *Cx30*^−/−^ cochlear lysates. **(E)** Histograms indicate optical density values (mean ± SEM) of the western blots normalized to tubulin levels. Full-scan Western blot images are shown in Supplementary Figure 5.

**Figure 4:**
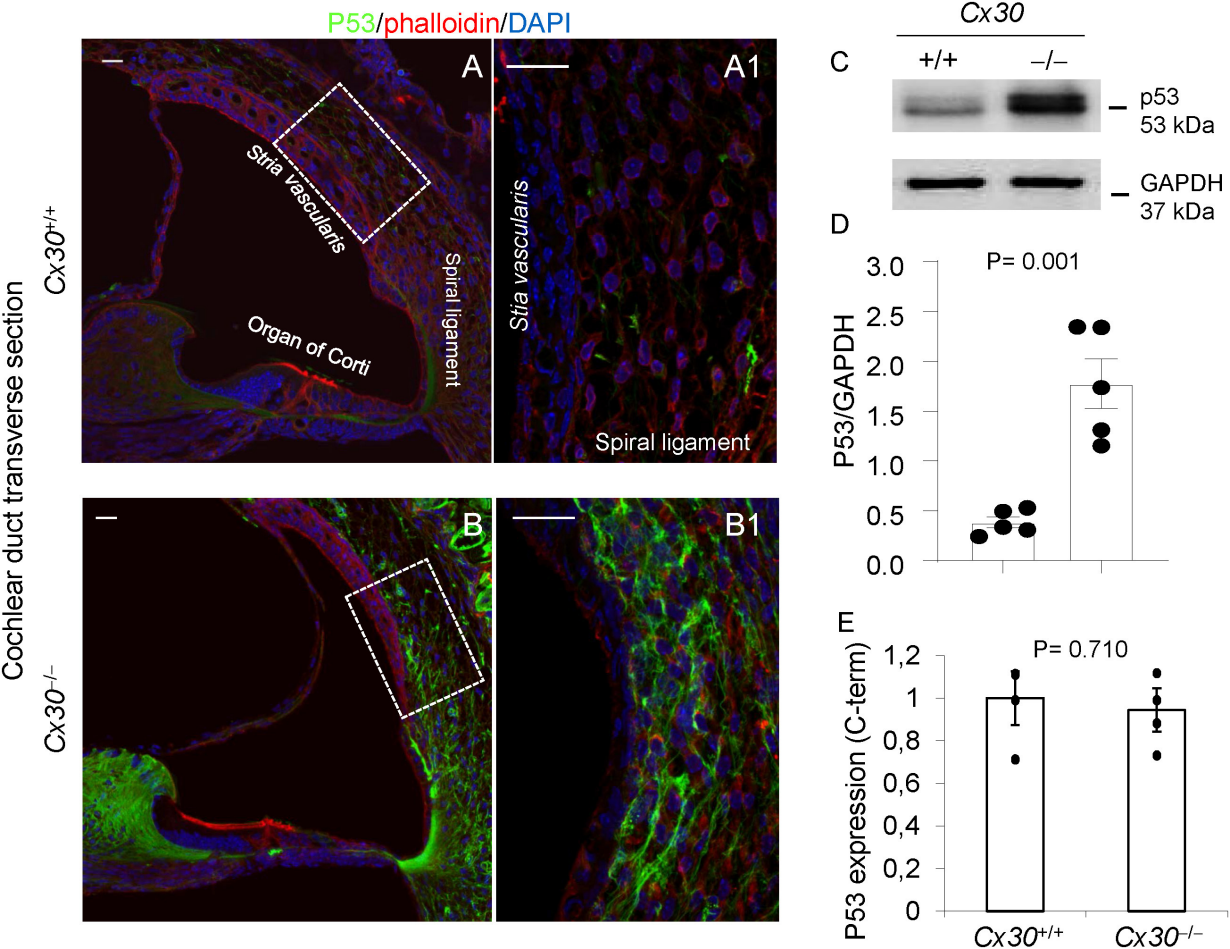
p53 expression in *Cx30*^+/+^ and *Cx30*^−/−^ cochleae at P5. **(A,B)** p53 immunoreactivity in the cochlear duct **(A,B)** and lateral wall (shown magnified in **A1,B1**) of *Cx30*^+/+^ and *Cx30*^−/−^ cochleae at P5. Scale bars: 20 μm. **(C)** Representative western blot immunoreactive bands quantifying the expression of p53 in *Cx30*^+/+^ and *Cx30*^−/−^ cochleae (P5). **(D)** Optical density analysis (mean ± SEM) normalized to GAPDH. **(E)** qPCR quantitative analysis (mean ± SEM) of cochlear p53 transcript levels in *Cx30*^+/+^ and *Cx30*^−/−^ mice. Full-scan western blot images are shown in Supplementary Figure 6.

To confirm the absence of Cx30 and drastically reduced levels of Cx26 in the cochleae of *Cx30*^−/−^ mice at P5, compared to age-matched *Cx30*^+/+^ controls (wt), we performed immunofluorescence studies (Supplementary Figure 3) and Western blotting (Supplementary Figure 4) with selective antibodies. The same approach was used to study the expression levels of *Sirt1* and *Tp53* protein products. Our results confirmed the downregulation of Sirt1 protein in the cochlear duct of *Cx30*^−/−^ mice, with the largest decrease observed in the *SV* and OC (Figure 3 and Supplementary Figure 5), suggesting that its miRNA-mediated inhibition can be post-transcriptionally regulated in our model. In addition, the elevated levels of miR-34c highlighted by expression profiling correlated with a dramatic increment of p53 immunoreactivity in *Cx30*^−/−^ cochleae (Figure 4 and Supplementary Figure 6), suggesting that the Sirt1–p53 axis was regulated downstream the post-transcriptional inhibition of *Sirt1* by upregulated miRNAs.

#### Analysis of Oxidative Stress in the Cochlea at P5

**Figure 5:**
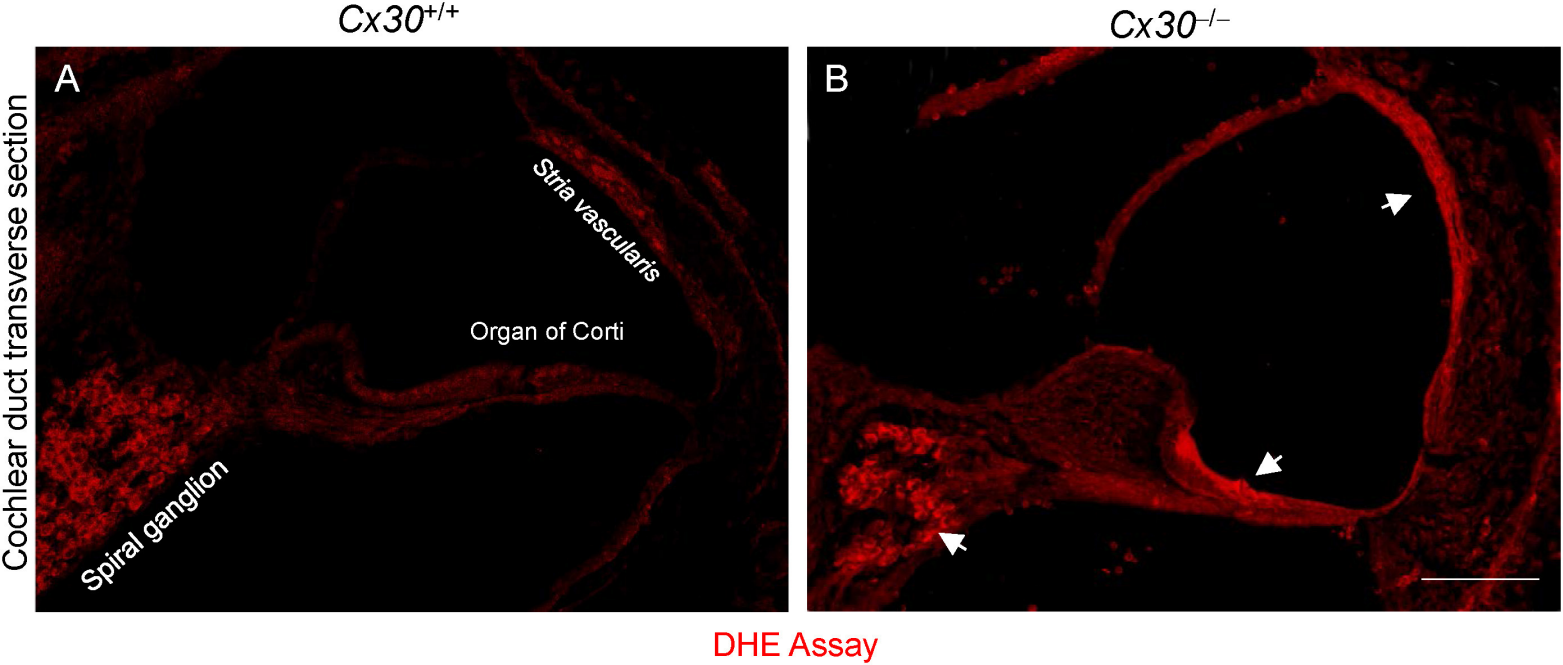
Oxidative stress in *Cx30*^+/+^ and *Cx30*^−/−^ cochleae at P5. **(A,B)** Representative cochlear cryosections stained with DHE showing superoxide production (red fluorescence) in *Cx30*^+/+^ and *Cx30*^−/−^ cochleae. Arrows indicate increased fluorescence intensity in principal cochlear structures: *Stria vascularis*, organ of Corti, and spiral ganglion. Scale bar: 100 μm.

As mentioned above, age-related cochlear hair cell apoptosis was linked to miR-34a/Sirt1/p53 action in a mouse model of ARHL (Xiong et al., 2015). It is well-known that oxidative stress is an aging hallmark and that an age-associated increase in oxidative stress reduces endothelial Sirt1 protein expression (Das et al., 2018). Therefore, we assayed oxidative stress levels in our model as previously done for conditional *Cx26*^−/−^ mice (Fetoni et al., 2018). In *Cx30*^+/+^ cochlear cryosections, superoxide levels (probed by DHE fluorescence, see Materials and Methods) were generally faint and slightly higher in spiral ganglion neurons (Figure 5A). In *Cx30*^−/−^ cochleae, superoxide levels were markedly higher and particularly evident in spiral ganglion neurons, OC, and *SV* (Figure 5B). Together, these data signal the occurrence of early oxidative damage in *Cx30*^−/−^ cochleae during post-natal development.

#### Analysis of Vascular Dysfunction in the *SV* at 3m

**Figure 6:**
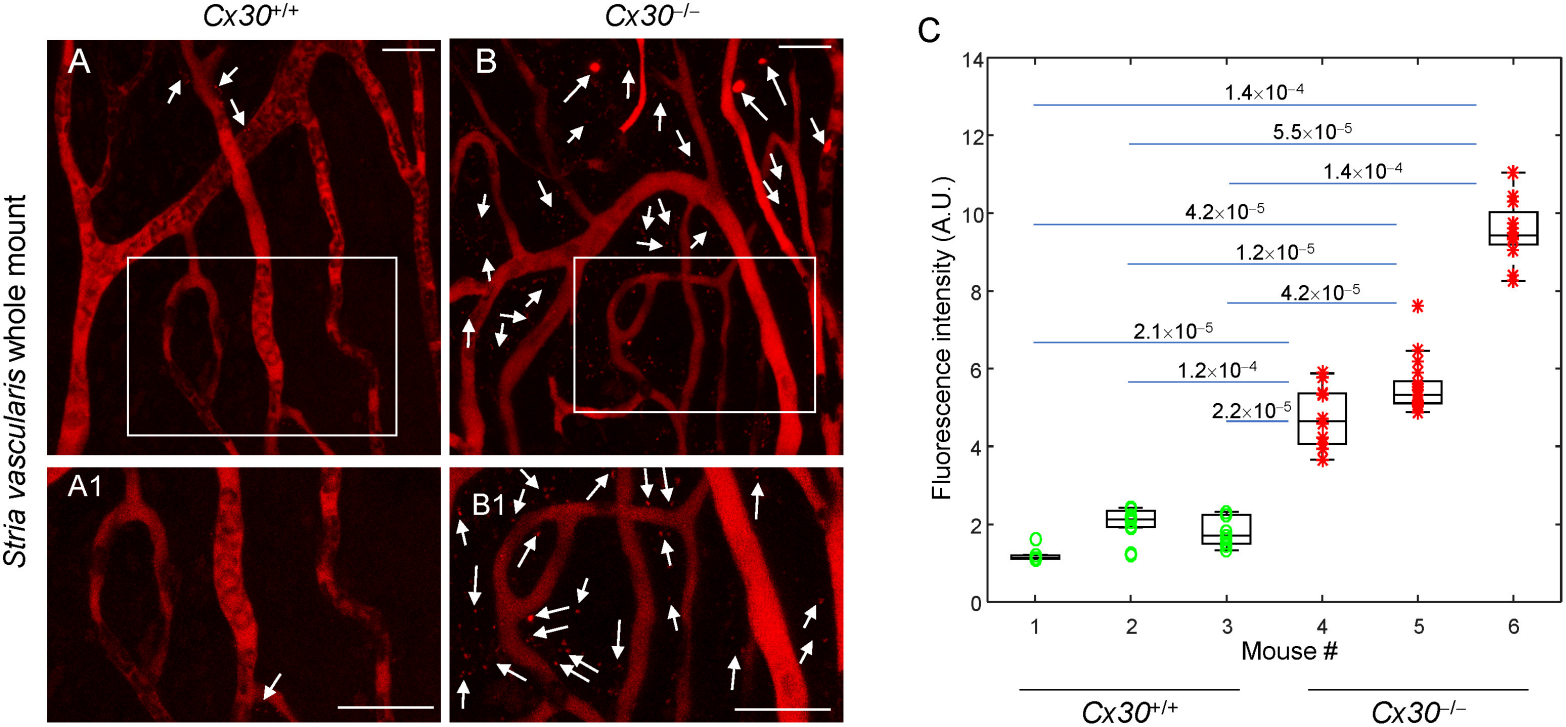
Vascular dysfunction in *Cx30*^−/−^ adult mice. **(A,B)** Representative images of freshly explanted *SV* from *Cx30*^+/+^ and *Cx30*^−/−^ mice (3 months of age) injected with a fluorescent Texas Red dextran (MW 70,000). Regions in the boxed areas are shown magnified in **(A1,B1)**. Arrows indicate dye lumps that leaked out of the vessels *Cx30*^−/−^. Scale bars: 30 μm. **(C)** Quantification of Texas Red dextran fluorescence intensity (AU, arbitrary units) in *SV* of *Cx30*^+/+^ and *Cx30*^−/−^ samples. Data analysis was performed on *n* = 3 mice of each genotype; numbers above horizontal bars indicate *p*-values that were determined by the Mann–Whitney *U*-test, as implemented in the MATLAB function *ranksum*. Through-focus image sequences (z-stacks) corresponding to the images shown in this figure are provided as Supplementary Video 1 (*Cx30*^+/+^) and Supplementary Video 2 (*Cx30*^−/−^).

The *SV* has the highest aerobic metabolic rate of all cochlear structures, and its microvascular alterations contribute to age-related degeneration and progressive decline of auditory function. To examine the effects of oxidative damage on the *SV*, we injected Texas Red dextran via the caudal vein in *Cx30*^−/−^ mice (at 3m) and age-matched *Cx30*^+/+^ controls and examined dye fluorescence in *SV* whole mounts. Figure 6 shows confocal images captured a few minutes after dye injection. There was no sign of dye extravasation in control littermates (Figures 6A,A1), whereas red fluorescence puncta were detected outside *SV* capillaries in *Cx30*^−/−^ samples (see arrows in Figures 6B,B1), indicating disruption of the endothelial barrier in adulthood. Also, as shown in Figure 6C, quantitative analysis of extravasation revealed a significant increase of Texas Red dextran fluorescence emission in the extravascular areas of *SV* of *Cx30*^−/−^ mice compared to age-matched *Cx30*^+/+^ controls.

## Discussion

In this work, we profiled miRNA and mRNA expression in the developing cochlea of a DFNB1 mouse model with global deletion of *Cx30* and severely reduced *Cx26* expression. Our results highlight an early oxidative stress that, later on, culminates in damage of the *SV*, a crucial vascularized epithelium of the cochlear lateral wall. The key protein Sirt1 (a NAD-dependent deacetylase protein) could mediate these processes, triggered by a lack of connexins. Our data suggest that Sirt1 downregulation could be potentially induced by miRNA negative influence and that it exerts its functions through p53, underlying hearing impairment.

The reduction of EP caused by *SV* dysfunction is responsible for strial ARHL in humans, as well as in mouse models of strial presbycusis (Keithley, 2019; Ohlemiller, 2019). EP reduction followed by mild hearing loss also affects a mouse model of digenic heterozygous deficiency of *Cx26* and *Cx30*, which causes impairment of heterotypic intercellular gap junctions coupling in the cochlear later wall (Mei et al., 2017). A recent study on *Cx30*^−/−^ mice (the same strain used in this article) confirmed lack of EP at P18 preceded by failure of mitochondrial function and ATP synthesis, increment of oxidative stress, and dysregulated expression of proteins required for EP generation in *SV*. That study also showed a significant increment of the pro-apoptotic proteins Bax, Bad, and caspase-3 in the mouse cochlea, suggesting a Bax-mediated mitochondrial cell death from P18 onward (Chen et al., 2020a). Data supporting the importance of *SV* leakage underlying the pathogenic process of hearing impairment are emerging also in a mouse model with conditional deletion of *Cx43* (Zhang et al., 2020).

We identified five overexpressed miRNAs (miR-34c, miR-29b, miR-29c, miR-141, and miR-181a) linked to apoptosis, degeneration of cochlea during aging and oxidative stress, with *Sirt1* as a common gene target of transcriptional and/or post-transcriptional regulation (Figure 1 and Table 1). Although *Sirt1* mRNA steady-state levels were not altered in *Cx30*^−/−^ samples, our immunoassays confirmed an early Sirt1 expression decrement in the cochleae of *Cx30*^−/−^ mice compared to controls. A miRNA-Sirt1 post-transcriptional influence has been validated in specific models (Zovoilis et al., 2011; Zhou et al., 2012; Bae et al., 2014). Therefore, *Sirt1* could be post-transcriptionally regulated by upregulated miRNAs, probably miR-34c, miR-29c, and miR-181a, in the cochleae of *Cx30*^−/−^ mice at P5. In particular, miR-34c is our best candidate, as our data suggest it could regulate the expression of *Cx26* (transcriptionally) and of *Sirt1* (post-transcriptionally). Moreover, miR-34a, p53 acetylation, and apoptosis increase with aging in the cochleae of an ARHL mouse model, together with an age-related decrement of Sirt1, linking the miR-34a/Sirt1/p53 axis to age-related apoptosis of cochlear cells (Xiong et al., 2015). As previously mentioned, miR-34c shares an identical seed sequence with miR-34a, suggesting they can have the same targets (Rokavec et al., 2014). In fact, miR-34c/Sirt1 post-transcriptional negative regulation was also described in a mouse model of cognitive decline (Zovoilis et al., 2011). Another work correlated age-dependent decrement of Sirt1 with increased miR-34a levels in the cochlea of an ARHL mouse model, accompanied by elevated hearing thresholds and loss of hair cells in the auditory cortex (Pang et al., 2016). The circulating plasma level of miRNA-34a was also significantly increased in human patients affected by ARHL (Pang et al., 2016). Moreover, increased levels of miR-34a were detected during endothelial cell senescence in an *in vitro* model and in older mice, and the effect of miR-34a upon senescence in endothelial cells was mediated by Sirt1 (Ito et al., 2010). It was also found that miR-34a levels increase during aging while Sirt1 levels decrease in murine aortas, and these changes correlated with an increased percentage of the senescence marker p16 and with miR-34a/Sirt1-mediated arterial dysfunctions (Badi et al., 2015). A correlation between SIRT1, Nrf2 and ROS production and their modulation by miR-34a in noise-induced hearing loss was reported also by Miguel et al. (2018).

Of note, Sirt1 was found abundantly expressed in the inner hair cells, strial marginal cells, and strial intermediate cells and moderately expressed in the outer hair cells and neurons of the auditory cortex and its significant reduction in a mouse model of ARHL correlated with elevated hearing thresholds and hair cell loss during aging (Xiong et al., 2014). In addition, the antiaging effect of a serotonin 5-HT3 receptor antagonist was assessed in a mouse model of induced senescence, resulting in the upregulation of *Sirt1* levels, increase of reduced glutathione concentration, decrease of inflammation biomarkers, increase of *Bcl-2*, and decrease of *Bax*, suggesting the regulation of oxidative stress, inflammation, and apoptosis was mediated by Sirt1 (Mirshafa et al., 2020).

The role of glutathione is particularly interesting, as we detected the downregulation of the potent antioxidant enzyme glutathione synthetase, *Gss* (Supplementary Table 1), also in a *Cx26* conditional knockout strain, accompanied by reduced release of glutathione through connexin hemichannels. In that work, we provided evidence of the consequent apoptosis and oxidative damage in the cochlear duct, offering a link between Cx26 monogenic hearing loss and ARHL (Fetoni et al., 2018). Moreover, the potent natural antioxidant and anti-inflammatory resveratrol is known to act on Sirt1, with positive antiaging effects on both brain (Sarubbo et al., 2017; Gomes et al., 2018) and vasculature (Kida and Goligorsky, 2016), also protecting vascular endothelial cells from atherosclerosis (a chronic inflammatory process associated with endothelial dysfunction and oxidative stress) (Wu et al., 2020). An oxidative stress increment in the *SV* was detected at P10 in *Cx30*^−/−^ mice, along with a deregulated expression of genes encoding for catalases involved in oxidative stress in different cochlear regions and time points (Chen et al., 2020a), in accord with the results reported here.

Our immunoassay data indicate that Sirt1 decrement is significant in *SV* at P5 (Figure 3), and Sirt1 is also known as a regulator of angiogenic signaling during blood vessel growth, downregulating genes involved in vascularization (Potente et al., 2007). Here, we also reported the early downregulation of *Ang, Ang4*, and *Ang6* (Supplementary Table 6). Our data also showed the deregulation of the trans-sulfuration pathway (Supplementary Tables 2, 6), which is part of the homocysteine metabolism upstream of glutathione synthesis (Ducker and Rabinowitz, 2017; Sbodio et al., 2019). Here, confirmed the downregulation of *Bhmt*, which was previously linked to elevated levels of homocysteine in the *SV* of *Cx30*^−/−^ mice (Cohen-Salmon et al., 2007), and of *Mthfr*, another gene involved in homocysteine metabolism and hearing loss, even if its role in ARHL is controversial. Interestingly, hearing loss has been linked also to nutritional imbalance and oxidative stress, promoting the use of dietary supplementation as a nutritional therapy (Partearroyo et al., 2017).

As noted above, Sirt1 is known to prevent oxidative damage through different mechanisms, including the regulation of mitochondrial dysfunction and oxidative stress by p53 and NRF2 that in turn control the glutathione pathway (Singh et al., 2018). We detected an early (i) relevant increment of p53 immunoreactivity in the cochlear lateral wall (Figure 4), (ii) oxidative stress damage concentrated in the *SV* at P5 (Figure 5) and culminating in an extravasation phenomenon in strial explants of young adult *Cx30*^−/−^ mice (Figure 6), and as previously discussed, (iii) the downregulation of *Gss* (Supplementary Table 1). Of note, our previous work on a Cx26 conditional knockout mouse model highlighted accelerated presbycusis caused by redox imbalance and dysregulation of Nrf2 antioxidant-response element-dependent genes related to glutathione metabolism (Fetoni et al., 2018). Moreover, Nrf2 seems to be also connected to the trans-sulfuration pathways (Sbodio et al., 2019).

**Figure 7:**
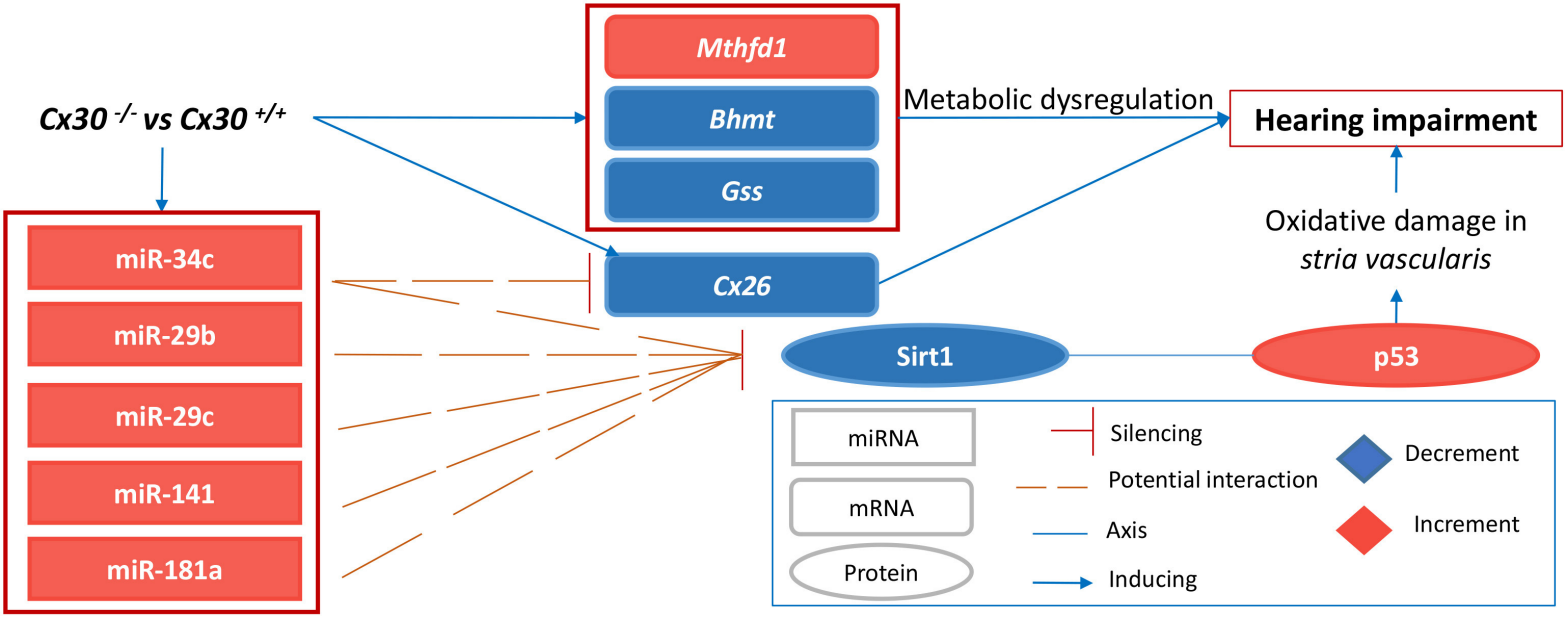
Pathogenic mechanism proposed. The scheme describes our results in a mouse model of inherited digenic deafness at P5, highlighting an early involvement of a miRNA-mediated Sirt1–p53 axis as a nexus between oxidative stress in *SV*, metabolic dysregulation, and a hearing impairment occurring later in time with vascular dysfunction, which are all hallmarks of aging.

In support of the involvement of p53, encoded by the *Trp53* gene, downstream of Sirt1, we also identified p53 as a hub of the PPI network built using all the deregulated miRNA targets of our model (Figure 2 and Supplementary Tables 4, 5). p53 is known to be a deacetylation target of Sirt1, exerting its pro-aging activity through the inhibition of DNA damage and stress-mediated cellular senescence. Consequently, inhibition of p53 by activation of SIRT1 has been proposed as a potential therapeutic strategy for aging-related diseases (Chen et al., 2020b). p53 regulates caspase-mediated apoptosis through two main mechanisms (and their cross talk): one is the extrinsic receptor-mediated pathway, acting via a transcription-dependent activity; the other is a transcription-independent activity that promotes mitochondrial outer membrane permeability (Speidel, 2010). As for the latter, wild-type p53 has been shown to localize at the endoplasmic reticulum (ER) and mitochondria-associated membranes (MAMs), where it interacts with sarco/ER Ca^2+^-ATPase (SERCA) pumps, modulating ER–mitochondria cross talk and, in turn, Ca^2+^-dependent apoptosis (Giorgi et al., 2015). In particular, activation and accumulation of p53 at the ER/MAMs render cells more prone to death, whereas absence of p53 leads to lower steady-state levels of reticular Ca^2+^, reduced Ca^2+^ mobilization, and mitochondrial accumulation evoked by agonist stimulation (ATP) or oxidative stress (Giorgi et al., 2016). In support of the role of p53 in our model, we detected a relevant upregulation of the *Tnfrsf10b* at P5 (Supplementary Table 6), which encodes a death receptor also known as Dr5 that is a target of the transcription-dependent apoptotic activity of p53 (Speidel, 2010). Moreover, we already discussed a recent report suggesting that high levels of ROS may promote a Bax-mediated mitochondrial cell death from P18 onward in a *Cx30*^−/−^ mouse model (Chen et al., 2020a). Taken together, these data suggest the involvement of the Sirt1–p53 axis as a nexus between (so far unrelated observations underlying) hearing impairment, vascular dysfunction, and aging in a mouse model of inherited digenic deafness (Figure 7).

In summary, our results suggest that inner ear connexin dysfunction in the *Cx30*^−/−^ mouse model of DFNB1 promotes oxidative stress, apoptosis, and degeneration during early post-natal development, ensuing in cochlear vascular dysfunction later on in life, which are all hallmarks of ARHL. Our analysis links these pathological modifications to a Sirt1–p53 axis and its possible miRNA regulation. Strial atrophy is a known cause of sensorineural hearing loss (Pauler et al., 1988), and the crucial role of ion flow in *SV* and the production and regulation of cochlear endolymph and the endolymphatic potential is well-established. Moreover, studies in different rodent species showed age-related strial degeneration, coupled with an age-related reduction in the endolymphatic potential (Fetoni et al., 2011; Kujawa and Liberman, 2019). Furthermore, recent work on patients affected by severe ARHL (both sporadic and familial cases) demonstrated that ultrarare gene variants are causally linked to presbycusis where they were already known to cause dominant early-onset monogenic deafness (Boucher et al., 2020). In addition, Cx26 and Cx30 gap junctions are known to connect intermediate and basal cells of cochlear *SV* and play an important role in the generation of the endocochlear potential (Wangemann, 2006). It remains to be determined whether the observed vascular dysfunction is caused by reduction of Cx26 alone or in combination with Cx30. Further analysis is also necessary to extend our integrative analysis and directly validate miRNA/mRNA interaction. Although such studies pose important experimental challenges in the auditory organ, future experiments may help to identify novel therapeutic strategies.

## Dedication

This work is dedicated to the memory of our colleague Barbara Maino.

## Data Availability Statement

The datasets presented in this study can be found in online repositories. The names of the repository/repositories and accession number(s) can be found at: https://www.ncbi.nlm.nih.gov/geo/query/acc.cgi?acc=GSE151368; https://www.ncbi.nlm.nih.gov/geo/query/acc.cgi?acc=GSE151367.

## Author Contributions

AF, SC, and FM: conceptualization. AS and MG: data curation. FP, VZ, and AS: formal analysis. MR, AF, SC, and FM: funding acquisition. GG, VZ, MG, and GC: investigation. GG, FP, AT-M, MR, AF, SC, and FM: methodology. AF, SC, and FM: resources. FS, MR, AF, SC, and FM: supervision. FP and VZ: validation. FP, VZ, and AS: visualization. GG, FP, and FM: writing—original draft. AF, SC, and FM: writing—review and editing. All authors contributed to the article and approved the submitted version.

## Conflict of Interest

The authors declare that the research was conducted in the absence of any commercial or financial relationships that could be construed as a potential conflict of interest.

## Supplementary Material

Supplementary figures and tables can be found in Data Sheet 1 and 2 files, respectively.

## Abbreviations

ARHL: Age-related hearing loss
DEG: Differentially expressed gene
DFNB1: Non-syndromic hearing loss and deafness 1
ER: Endoplasmic reticulum
GO: Gene ontology
GEO: Gene Expression Omnibus
m: Post-natal month
MAM: Mitochondria-associated membrane
miRNA: MicroRNA
OC: Organ of Corti
P: Post-natal day
PPI: Protein–protein interaction
SERCA: Sarcoplasmic/endoplasmic reticulum calcium ATPase
*SV*: *Stria vascularis*.
