# Supplementary material for: miRNA and mRNA Profiling Links Connexin Deficiency to Deafness via Early Oxidative Damage in the Mouse Stria Vascularis
Source: Front Cell Dev Biol. 2021 Jan 25;8:616878. doi: 10.3389/fcell.2020.616878 (PMC7868390; doi:10.3389/fcell.2020.616878)
Supplement: Supplementary file 1 [file Data_Sheet_1.PDF]

SUPPLEMENTARY FIGURES

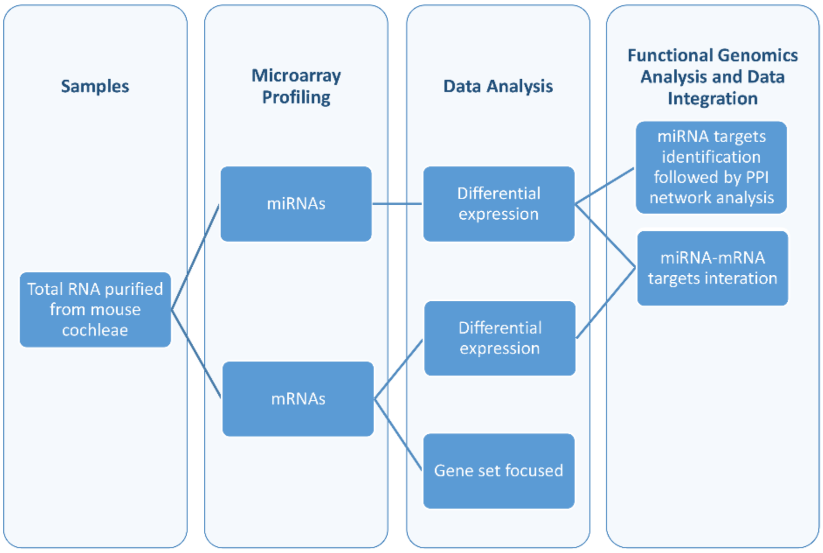

Figure S1. Data analysis flow chart. Methods used to analyse and integrate genomics data

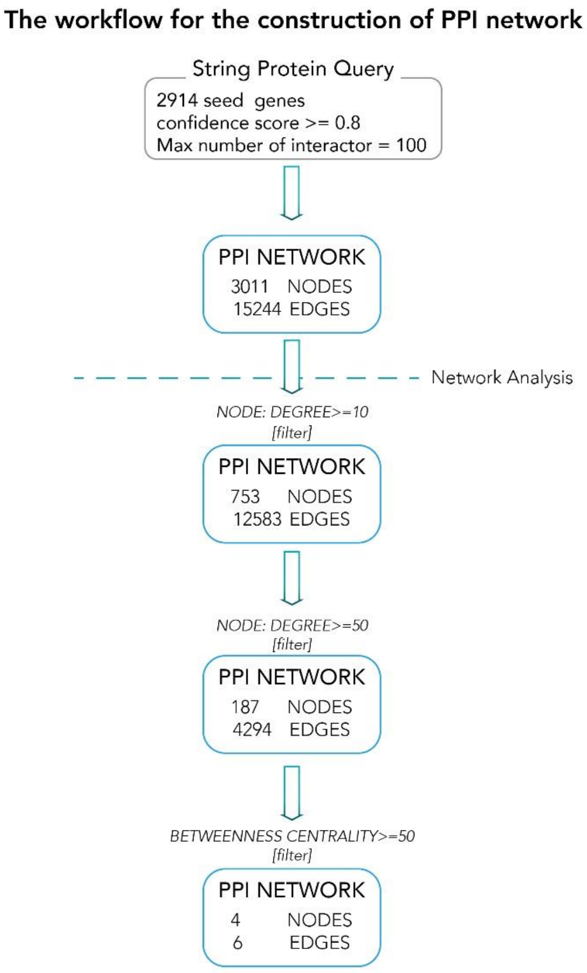

Figure S2. Workflow of PPI network construction and analysis. The workflow, setting and filter values used to obtain an extended PPI network, starting with 2914 target genes of the sixteen deregulated miRNAs as input data. Where nodes are proteins communicating in a biological network and edges indicate their interactions, while node degree and edge betweeness centrality represent topological parameters used to obtain hub nodes.

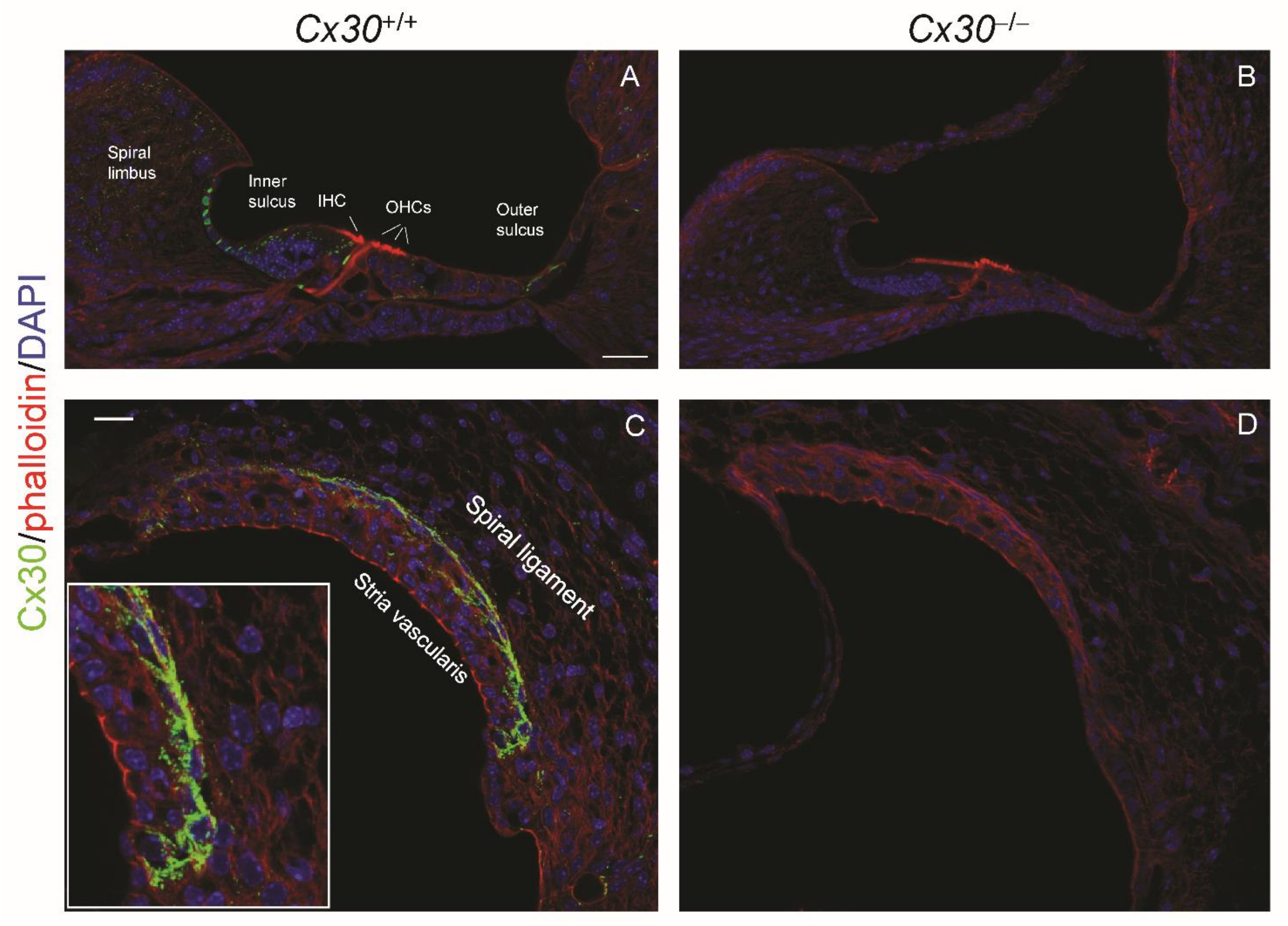

Figure S3. Connexin 30 localization in *Cx30*$^{+/+}$ and *Cx30*$^{-/-}$ cochleae at P5. A-D: Cx30 immunoreactivity in spiral limbus and sensory epithelium (A-B) lateral wall and stria vascularis (C-D) of *Cx30*$^{+/+}$ and *Cx30*$^{-/-}$ cochleae. Scale bar: 30 µm.

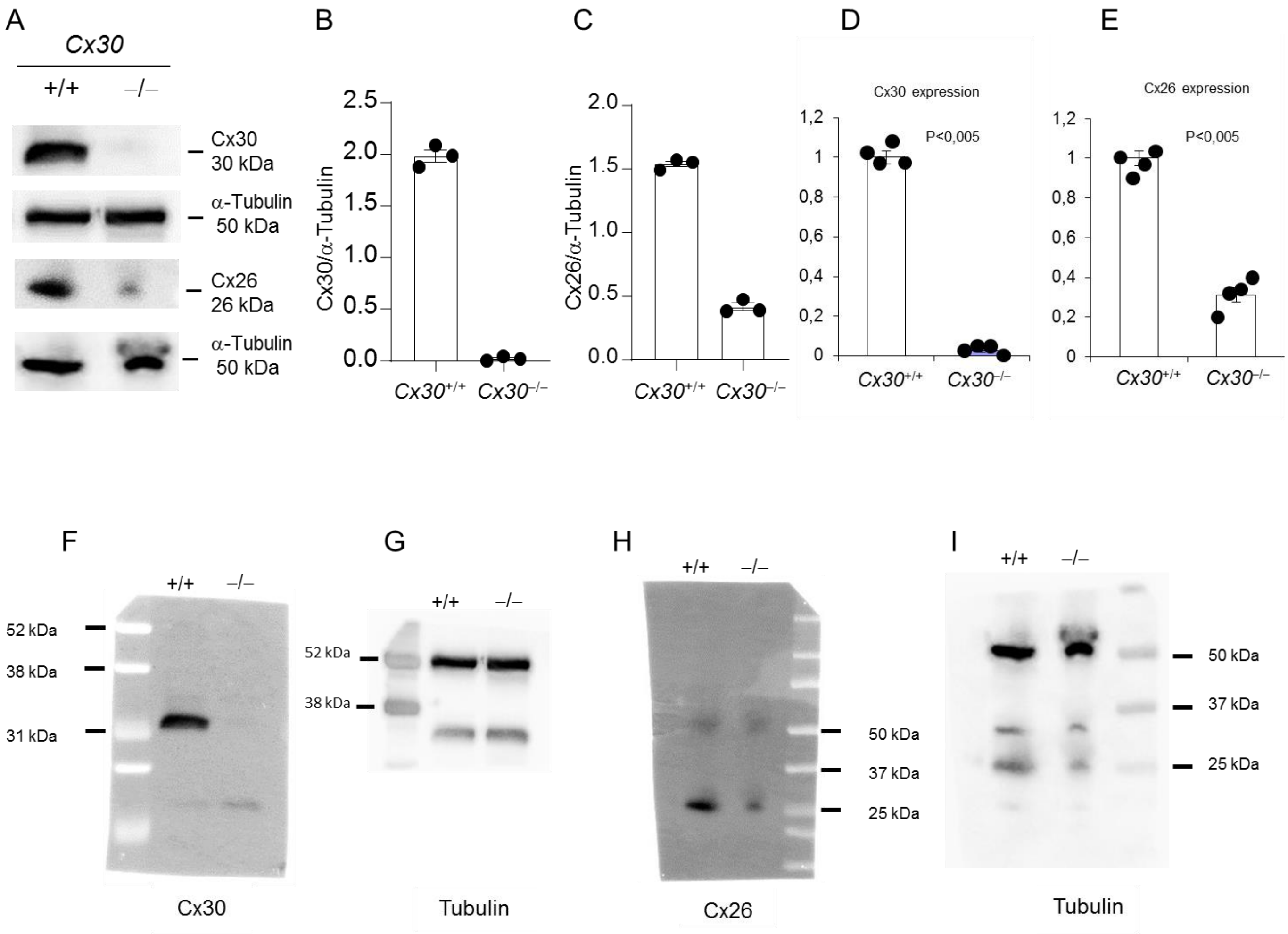

Figure S4. Cx30 and Cx26 expression in the cochlea of *Cx30*$^{+/+}$ and *Cx30*$^{-/-}$ mice at P5. A: Representative western blot immunoreactive bands showing the expression of Cx30 and Cx26 in *Cx30*$^{+/+}$ and *Cx30*$^{-/-}$ cochleae. B-C: Histograms (mean ± S.E.M.) represent optical density values normalized to α-tubulin levels. D-E: Histograms (mean ± S.E.M.) show qPCR quantitative analysis of cochlear Cx30 and Cx26 mRNA transcription in *Cx30*$^{+/+}$ and *Cx30*$^{-/-}$ cochleae. F-G: Uncropped western blot bands of western blot for Cx30 (F) and relative α-tubulin (G). H-I: Uncropped western blot bands of western blot for Cx26 (H) and relative α-tubulin (I).

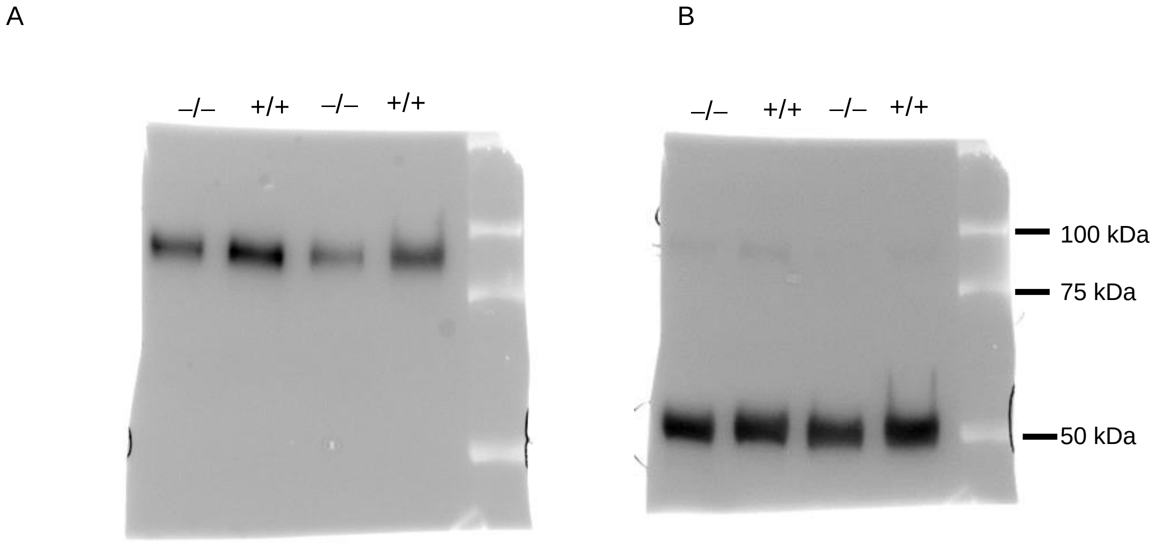

Figure S5. Uncropped western blot bands of western blot for Sirt1 (A) and α-tubulin (B), showed in Figure 3.

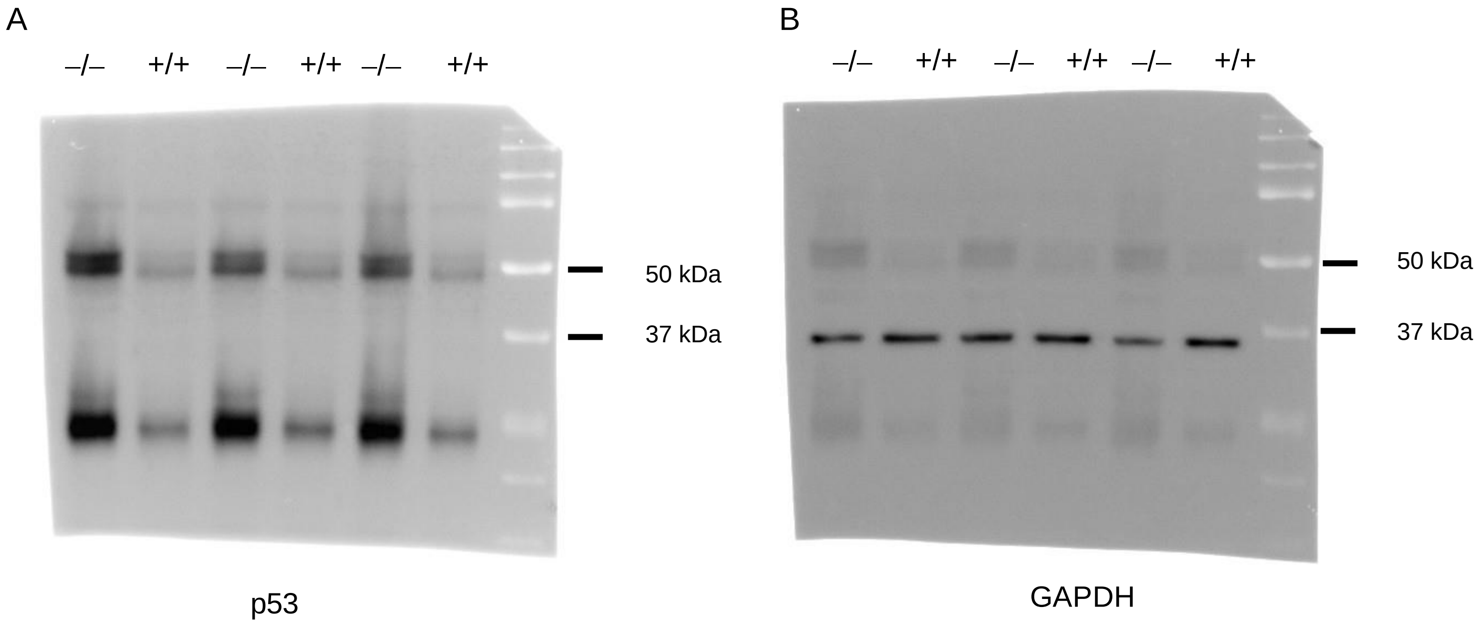

Figure S6. Uncropped western blot bands of western blot for p53 (A) and GAPDH (B), showed in Figure 4.
