# Supplementary material for: miRNA and mRNA Profiling Links Connexin Deficiency to Deafness via Early Oxidative Damage in the Mouse Stria Vascularis
Source: Front Cell Dev Biol. 2021 Jan 25;8:616878. doi: 10.3389/fcell.2020.616878 (PMC7868390; doi:10.3389/fcell.2020.616878)
Supplement: Supplementary file 2 [file Data_Sheet_2.pdf]

Supplementary Tables

Supplementary Table S1. Gene set-focused analysis of glutathione-related genes. Lists of glutathione-related genes and their expression values by moderate T-test.

GLUTHATIONE GENE LIST

**GeneSymbol**	
Abcc1	*Gstp1*
Adh4	*Gstp2*
Adh5	*Gstp3*
Aldh5a1	*Gstt1*
Alox5ap	*Gstt2*
Chac1	*Gstt3*
Chac2	*Gstt4*
Cth	*Gstz1*
Ctns	*Hagh*
Eif2ak3	*Haghl*
Ethe1	*Hmgn5*
G6pdx	*Hpgds*
Gclc	*Idh1*
Gclm	*Lancl1*
Ggt1	*Ltc4s*
Ggt5	*Mgst1*
Ggt6	*Mgst2*
Ggt7	*Mgst3*
Glo1	*Mmachc*
Glrx	*Nat8*
Glrx3	*Nfe2l1*
Glrx5	*Nfe2l2*
Gm10639	*Oplah*
Gm20441	*Park7*
Gm6665	*Pdia3*
Gpx1	*Pnkd*
Gpx2	*Prdx6*
Gpx3	*Ptges*
Gpx4	*Slc22a8*
Gpx5	*Slc7a11*
Gpx6	*Slc9a3r1*
Gpx7	*Snca*
Gpx8	*Sod1*
Gsr	*Sod2*
Gss	*Txndc12*
Gsta1	*Txnrd3*
Gsta2	
Gsta3	
Gsta4	
Gstcd	
Gstk1	
Gstm1	
Gstm2	
Gstm3	
Gstm4	
Gstm5	
Gstm6	
Gstm7	
Gsto1	
Gsto2	

GLUTHATIONE MODERATED T-TEST

ProbeName	p ([WT] vs [KO])	Regulation	FC (abs)	FC	Log FC	[WT](raw)	[KO](raw)	[WT](normalized)	[KO](normalized)	GeneSymbol	Description	Sequence
A_66_P136921	0,008074958	down	1,5418512	-1,5418512	-0,6246635	270,19623	171,72308	0,30325353	-0,32140994	***Gss***	Mus musculus glutathione synthetase (Gss), transcript variant 1, mRNA [NM_008180]	AGAGGGTGGAGGTAACAACTTATACGGGGAGGAAATGGTACAAGCTCTGGAGCAGCTGAA

Supplementary Table S2. Gene set-focused analysis of homocysteine-related genes. Lists of homocysteine-related genes and their expression values by moderate T-test.

HOMOCYSTEIN GENE LIST

GeneSymbol
Bhmt
Bhmt2
Blmh
Cbs
Comt
Cps1
Cth
Dpep1
Herpud1
Mmut
Mpst
Mthfd1
Mthfr
Mtr
Mtrr
Nox4
Tst

HOMOCYSTEIN MODERATED T-TEST

ProbeName	p ([WT] vs [KO])	Regulation	FC (abs)	FC	Log FC	[WT](raw)	[KO](raw)	[WT](normalized)	[KO](normalized)	GeneSymbol	Description	Sequence
A_55_P2740152	0,031401385	up	2,275023	2,275023	1,1858809	2468,771	5503,745	-0,9697015	0,21617937	***Mthfd1***	Mus musculus methylenetetrahydrofolate dehydrogenase (NADP+ dependent), methenyltetrahydrofolate cyclohydrolase, formyltetrahydrofolate synthase (Mthfd1), mRNA [NM_138745]	GAAGAGTACCACCACGATCGGGCTGGTGCAGGCCCTCGGCGCCCACCTGCGACAGAACGT
A_55_P2714597	0,002541643	down	2,057475	-2,057475	-1,040875	34,73928	16,545435	0,49040174	-0,5504731	***Bhmt***	Mus musculus betaine-homocysteine methyltransferase (Bhmt), mRNA [NM_016668]	ACAATGGACTGCTATTCTGTCTGCTTCTATTCCATTTCATTAATAAAAATGTGCTGGTTG

Supplementary Table S3. Deregulated miRNAs in *Cx30*$^{-/-}$

Deregulated miRNAs	FC[a]	Validated targets[b]	Predicted targets[c]
mmu-miR-18a-5p	12.624649	403	-
mmu-miR-19a-3p	35.45887	749	-
mmu-miR-29b-3p	17.24806	813	-
mmu-miR-29c-3p	51.16448	273	-
mmu-miR-34b-5p	16.681662	4	-
mmu-miR-34c-5p	16.490164	227	-
mmu-miR-141-3p	25.640936	65	-
mmu-miR-181a-1-3p	16.851034	-	183
mmu-miR-185-5p	6.8337	116	-
mmu-miR-218-5p	14.799809	531	-
mmu-miR-301a-3p	10.196878	590	-
mmu-miR-301b-3p	6.519308	578	-
mmu-miR-335-5p	10.260769	504	-
mmu-miR-376a-3p	24.383179	-	118
mmu-miR-872-5p	17.254332	4	-
mmu-miR-6997-5p	-3.505543	-	28

[a,b] Fold change (FC) and number of miRNAs validated targets contained in DIANA-TarBase v7.0 are indicated.

[c] For the three deregulated miRNAs with no validated gene targets, three tools for miRNA targets prediction have been queried as described in Materials and Methods section.

Supplementary Table S4. PPI network nodes and their topological features

For all entries: Species = mus musculus; Node Type = protein; NameSpace = stringdb.; Is Single Node = false; Number of Directed Edges = 0; Partner of MultiEdge Node Pairs = 0; Selected = false; Self Loops = 0.

SUID	Database Identifier	Canonical Name	Name	Query Term	Description	Betweenness Centrality	Average Shortest Path Length	Clustering Coefficient	Closeness Centrality	Degree	Eccentricity	Neighborhood Connectivity	Number of Undirected Edges	Radiality	Stress	Topological Coefficient
11212	10090.ENSMUSP00000136791	J3QK04	Gm7808		predicted pseudogene 7808	0.07577783	236.340.534	0.10805452	0.42311828	270	7	4.564.814.815	270	0.89512267	10281420	0.04686668
14030	10090.ENSMUSP00000125548	O70435	Psma3	Psma3	proteasome (prosome, macropain) subunit, alpha type 3	8,62E+00	283.481.576	0.54828797	0.35275661	68	8	6.932.352.941	68	0.85886033	392322	
11733	10090.ENSMUSP00000059289	Q9Z0W3	Nup160	Nup160	nucleoporin 160	0.00212911	301.143.583	0.52308591	0.33206751	59	8	6.430.508.475	59	0.84527417	539728	0.1509509
12511	10090.ENSMUSP00000099475	Q68FD5	Cltc	Cltc	clathrin, heavy polypeptide (Hc)	0.00424863	281.639.136	0.56704261	0.35506429	57	7	580.877.193	57	0.86027759	580594	
13798	10090.ENSMUSP00000079124	O70201	Birc5	Birc5	baculoviral IAP repeat-containing 5	0.00480047	285.895.807	0.44243133	0.34977778	59	8	5.403.389.831	59	0.85700323	526110	
12009	10090.ENSMUSP00000029201	P42337	Pik3ca	Pik3ca	phosphatidylinositol 3-kinase, catalytic, alpha polypeptide	0.00724354	280.177.891	0.26035232	0.3569161	94	8	4.607.446.809	94	0.86140162	983148	
11498	10090.ENSMUSP00000028599	Q99LI7	Cstf3	Cstf3	cleavage stimulation factor, 3' pre-RNA, subunit 3	7,02E+00	323.697.586	0.89384921	0.30893032	64	8	7.778.125	64	0.82792493	263226	0.25585938
11499	10090.ENSMUSP00000033609	Q8BIQ5	Cstf2	Cstf2	cleavage stimulation factor, 3' pre-RNA subunit 2	0.00223153	316.899.619	0.84055944	0.31555734	66	8	76.0	66	0.83315414	427720	0.22749955
11244	10090.ENSMUSP00000032399	P32883	Kras	Kras	v-Ki-ras2 Kirsten rat sarcoma viral oncogene homolog	0.00873019	267.153.748	0.24593838	0.37431629	85	8	5.821.176.471	85	0.87142019	1550574	
12270	10090.ENSMUSP00000037268	Q8VDM6	Hnrnpul1	Hnrnpul1	heterogeneous nuclear ribonucleoprotein U-like 1	3.2E-7	324.396.442	0.99774011	0.30826479	60	8	81.85	60	0.82738735	48	0.27102649
13551	10090.ENSMUSP00000021447	Q61151	Ppp2r5e	Ppp2r5e	protein phosphatase 2, regulatory subunit B (B56), epsilon isoform	0.00222653	272.236.341	0.47167716	0.36732789	74	8	7.406.756.757	74	0.86751051	683436	0.10550936
12016	10090.ENSMUSP00000035037	Q8BTI9	Pik3cb	Pik3cb	phosphatidylinositol 3-kinase, catalytic, beta polypeptide	0.00190236	291.041.931	0.33654202	0.3435931	74	8	4.966.216.216	74	0.85304467	513772	
13809	10090.ENSMUSP00000051092	Q921L0	Picalm	Picalm	phosphatidylinositol binding clathrin assembly protein	0.00101318	289.834.816	0.71510204	0.34502411	50	7	62.98	50	0.85397322	242648	
12018	10090.ENSMUSP00000114180	Q9ET24	Ubc	Ubc	ubiquitin C	0.05482875	240.025.413	0.12827787	0.41662255	235	7	4.778.297.872	235	0.89228814	8010914	0.04982584
13043	10090.ENSMUSP00000030417	Q8BQ51	Cdc42	Cdc42	cell division cycle 42	0.03597249	258.005.083	0.16	0.38758926	126	8	4.927.777.778	126	0.87845763	3695196	
13047	10090.ENSMUSP00000097547	P39688	Fyn	Fyn	Fyn proto-oncogene	0.00895724	277.890.724	0.2436036	0.35985368	75	7	53.28	75	0.86316098	1113208	0.08689505
11263	10090.ENSMUSP00000021046	Q810A7	Ddx42	Ddx42	DEAD (Asp-Glu-Ala-Asp) box polypeptide 42	8,97E-02	324.205.845	0.94870439	0.30844601	62	8	7.975.806.452	62	0.82753397	65802	0.26322794
11265	10090.ENSMUSP00000133245	Q569Z5	Ddx46	Ddx46	DEAD (Asp-Glu-Ala-Asp) box polypeptide 46	8,97E-02	324.205.845	0.94870439	0.30844601	62	8	7.975.806.452	62	0.82753397	65802	0.26322794
12296	10090.ENSMUSP00000067786	Q62245	Sos1	Sos1	son of sevenless homolog 1 (Drosophila)	7,08E+00	304.193.139	0.38588235	0.32873851	51	8	5.531.372.549	51	0.84292835	95034	
12043	10090.ENSMUSP00000020085	P61080	Ube2d1	Ube2d1	ubiquitin-conjugating enzyme E2D 1	0.00125841	29.866.582	0.48838897	0.33482238	53	8	6.877.358.491	53	0.84718014	176542	
12047	10090.ENSMUSP00000018685	Q9DBF7	Cwc25	Cwc25	CWC25 spliceosome-associated protein homolog (S. cerevisiae)	3.2E-7	324.396.442	0.99774011	0.30826479	60	8	81.85	60	0.82738735	48	0.27102649
14097	10090.ENSMUSP00000001818	P63154	Crnkl1	Crnkl1	Crn, crooked neck-like 1 (Drosophila)	1,07E+00	323.697.586	0.92780338	0.30893032	63	8	7.885.714.286	63	0.82792493	69584	0.25854801
11539	10090.ENSMUSP00000047865	Q80X98	Dhx38	Dhx38	DEAH (Asp-Glu-Ala-His) box polypeptide 38	6,58E+00	323.697.586	0.92421915	0.30893032	63	8	7.903.174.603	63	0.82792493	183652	0.25912048
13588	10090.ENSMUSP00000070726	Q6PD03	Ppp2r5a	Ppp2r5a	protein phosphatase 2, regulatory subunit B (B56), alpha isoform	0.00289847	271.664.549	0.44019139	0.36810103	77	8	7.211.688.312	77	0.86795035	826016	0.10214856
11796	10090.ENSMUSP00000002198	Q8K4Z5	Sf3a1	Sf3a1	splicing factor 3a, subunit 1	9,78E-02	324.142.313	0.92524322	0.30850647	63	8	7.884.126.984	63	0.82758284	67270	0.26020221
12309	10090.ENSMUSP00000090059	Q62093	Srsf2	Srsf2	serine/arginine-rich splicing factor 2	4,99E+00	324.269.377	0.94606029	0.30838558	62	8	7.996.774.194	62	0.82748509	108198	0.26479385
12310	10090.ENSMUSP00000117045	P84104	Srsf3	Srsf3	serine/arginine-rich splicing factor 3	6.85E-6	32.433.291	0.97704918	0.30832517	61	8	812.295.082	61	0.82743622	1982	0.26897188
13592	10090.ENSMUSP00000002839	Q91V89	Ppp2r5d	Ppp2r5d	protein phosphatase 2, regulatory subunit B (B56), delta isoform	0.0028866	269.631.512	0.45684211	0.37087653	76	8	7.447.368.421	76	0.86951422	981326	
12570	10090.ENSMUSP00000003310	Q9ERU9	Ranbp2	Ranbp2	RAN binding protein 2	0.01282091	285.895.807	0.30935731	0.34977778	78	7	5.326.923.077	78	0.85700323	1371314	
12572	10090.ENSMUSP00000109635	Q9Z0R4	Itsn1	Itsn1	intersectin 1 (SH3 domain protein 1A)	0.00264335	278.907.243	0.57982583	0.35854214	53	8	679.245.283	53	0.86237904	361160	
12574	10090.ENSMUSP00000028475	Q99LI9	Clp1	Clp1	CLP1, cleavage and polyadenylation factor I subunit	2,05E+00	324.269.377	0.94764675	0.30838558	62	8	8.001.612.903	62	0.82748509	108362	0.26495407
12319	10090.ENSMUSP00000119065	Q3TWW8	Srsf6	Srsf6	serine/arginine-rich splicing factor 6	5,67E+00	323.761.118	0.91909882	0.3088697	63	8	789.047.619	63	0.82787606	212620	0.25955514
13088	10090.ENSMUSP00000028278	O35593	Psmd14	Psmd14	proteasome (prosome, macropain) 26S subunit, non-ATPase, 14	0.00140953	283.290.978	0.50422535	0.35299394	71	8	668.028.169	71	0.85900694	486442	0.11060069
12325	10090.ENSMUSP00000120595	Q6PDM2	Srsf1	Srsf1	serine/arginine-rich splicing factor 1	3,10E+00	324.269.377	0.94711793	0.30838558	62	8	7.998.387.097	62	0.82748509	163358	0.26484725
14119	10090.ENSMUSP00000053897	P70424	Erbb2	Erbb2	v-erb-b2 erythroblastic leukemia viral oncogene homolog 2	0.00347836	276.111.817	0.37330317	0.36217211	52	8	6.946.153.846	52	0.86452937	859710	0.10398434
11562	10090.ENSMUSP00000098066	Q6ZWM3	Actb	Actb	actin, beta	0.02938551	27.090.216	0.14475743	0.36913696	72	8	4.055.555.556	72	0.8685368	3243140	
12842	10090.ENSMUSP00000031695	Q91YD9	Wasl	Wasl	Wiskott-Aldrich syndrome-like (human)	0.00336312	277.001.271	0.49727768	0.36100917	58	8	6.324.137.931	58	0.86384518	472900	
13355	10090.ENSMUSP00000068487	Q62313	Tgoln1	Tgoln1	trans-golgi network protein	0.00393259	289.008.895	0.58333333	0.34601011	57	7	5.764.912.281	57	0.85460854	615888	0.10481659
11827	10090.ENSMUSP00000025705	Q62120	Jak2	Jak2	Janus kinase 2	0.00509007	301.016.518	0.28470588	0.33220768	51	7	4.503.921.569	51	0.84537191	618520	
13878	10090.ENSMUSP00000099790	P42567	Eps15	Eps15	epidermal growth factor receptor pathway substrate 15	0.00647547	287.865.311	0.61306122	0.34738468	50	8	62.22	50	0.85548822	488094	
12353	10090.ENSMUSP00000026416	P97377	Cdk2	Cdk2	cyclin-dependent kinase 2	0.00643497	275.349.428	0.32534247	0.3631749	73	8	5.947.945.205	73	0.86511582	744922	
11334	10090.ENSMUSP00000030940	P62874	Gnb1	Gnb1	guanine nucleotide binding protein (G protein), beta 1	0.00353346	299.491.741	0.29632653	0.33389902	50	8	43.92	50	0.84654481	608088	
11335	10090.ENSMUSP00000068148	Q9QXK7	Cpsf3	Cpsf3	cleavage and polyadenylation specificity factor 3	0.00167511	314.104.193	0.81908639	0.3183657	67	8	7.591.044.776	67	0.83530447	411886	0.21688699
12359	10090.ENSMUSP00000088845	Q03147	Cdk7	Cdk7	cyclin-dependent kinase 7	0.00422194	28.678.526	0.39464442	0.34869296	68	7	6.811.764.706	68	0.85631903	575048	
11336	10090.ENSMUSP00000047797	O35218	Cpsf2	Cpsf2	cleavage and polyadenylation specific factor 2	0.00167511	314.104.193	0.81908639	0.3183657	67	8	7.591.044.776	67	0.83530447	411886	0.21688699
11338	10090.ENSMUSP00000038958	Q8BTV2	Cpsf7	Cpsf7	cleavage and polyadenylation specific factor 7	6.85E-6	32.433.291	0.97704918	0.30832517	61	8	812.295.082	61	0.82743622	1982	0.26897188
13389	10090.ENSMUSP00000030014	Q3UYV9	Ncbp1	Ncbp1	nuclear cap binding protein subunit 1	0.00437275	284.879.288	0.47527473	0.35102587	105	7	6.654.285.714	105	0.85778516	888690	
13647	10090.ENSMUSP00000039269	Q96J62	Hnrnpk	Hnrnpk	heterogeneous nuclear ribonucleoprotein K	3.2E-7	324.396.442	0.99774011	0.30826479	60	8	81.85	60	0.82738735	48	0.27102649
12880	10090.ENSMUSP00000090177	A2A5Z6	Smurf2	Smurf2	SMAD specific E3 ubiquitin protein ligase 2	0.00293942	304.574.333	0.47371032	0.32832708	64	8	595.625	64	0.84263513	557410	0.14559138
13649	10090.ENSMUSP00000132735	Q60668	Hnrnpd	Hnrnpd	heterogeneous nuclear ribonucleoprotein D	0.0021145	300.190.597	0.67053999	0.33312169	77	7	8.619.480.519	77	0.84600723	509566	0.17808844
11858	10090.ENSMUSP00000023486	Q62351	Tfrc	Tfrc	transferrin receptor	0.00298658	289.390.089	0.66138763	0.34555434	52	7	60.75	52	0.85431532	289832	0.11085766
13652	10090.ENSMUSP00000107237	Q9Z204	Hnrnpc	Hnrnpc	heterogeneous nuclear ribonucleoprotein C	0.00414794	310.800.508	0.78028169	0.3217498	71	7	75.0	71	0.83784576	469254	0.20321386
11351	10090.ENSMUSP00000024599	Q07113	Igf2r	Igf2r	insulin-like growth factor 2 receptor	0.00168571	289.580.686	0.67043741	0.3453269	52	7	6.159.615.385	52	0.8541687	344664	
13400	10090.ENSMUSP00000071200	P08775	Polr2a	Polr2a	polymerase (RNA) II (DNA directed) polypeptide A	0.01129392	270.965.693	0.36292063	0.36905041	126	7	6.217.460.317	126	0.86848793	1919144	0.09102001
13403	10090.ENSMUSP00000025106	Q9D7M8	Polr2d	Polr2d	polymerase (RNA) II (DNA directed) polypeptide D	0.00558795	278.716.645	0.39269972	0.35878733	121	7	6.372.727.273	121	0.86252566	1434926	0.10278592
13408	10090.ENSMUSP00000021277	O70126	Aurkb	Aurkb	aurora kinase B	0.00533417	281.321.474	0.40538786	0.35546522	79	8	6.103.797.468	79	0.86052194	828892	
11879	10090.ENSMUSP00000062392	P20444	Prkca	Prkca	protein kinase C, alpha	0.01475222	289.771.283	0.15912519	0.34509976	52	8	45.5	52	0.85402209	2892732	0.08990861
11113	10090.ENSMUSP00000099587	P63087	Ppp1cc		protein phosphatase 1, catalytic subunit, gamma isoform	0.00676045	29.815.756	0.40808081	0.33539314	55	8	4.876.363.636	55	0.84757111	764734	
11114	10090.ENSMUSP00000019614	Q9DCD2	Xab2		XPA binding protein 2	0.00133821	294.218.551	0.76445967	0.33988339	73	8	8.446.575.342	73	0.85060111	427008	0.15847233
11115	10090.ENSMUSP00000021412	Q9QUM9	Psma6		proteasome (prosome, macropain) subunit, alpha type 6	8,62E+00	283.481.576	0.54828797	0.35275661	68	8	6.932.352.941	68	0.85886033	392322	
11116	10090.ENSMUSP00000115883	Q00731	Vegfa		vascular endothelial growth factor A	0.010165	276.683.609	0.28156749	0.36142365	53	8	5.011.320.755	53	0.86408953	1000710	0.07913798
11117	10090.ENSMUSP00000103483	B1AWD9	Clta		clathrin, light polypeptide (Lca)	0.00289664	281.575.604	0.60808081	0.3551444	55	7	5.998.181.818	55	0.86032646	488714	0.09753141
11118	10090.ENSMUSP00000021063	Q9D8W5	Psmd12		proteasome (prosome, macropain) 26S subunit, non-ATPase, 12	8,62E+00	283.481.576	0.54828797	0.35275661	68	8	6.932.352.941	68	0.85886033	392322	0.11477406
11375	10090.ENSMUSP00000018449	Q99PV0	Prpf8	Prpf8	pre-mRNA processing factor 8	0.00141218	323.506.989	0.84801865	0.30911233	66	8	7.537.878.788	66	0.82807155	444624	0.24714357
11119	10090.ENSMUSP00000017365	P14685	Psmd3		proteasome (prosome, macropain) 26S subunit, non-ATPase, 3	8,62E+00	283.481.576	0.54828797	0.35275661	68	8	6.932.352.941	68	0.85886033	392322	0.11477406
11120	10090.ENSMUSP00000022380	P62334	Psmc6		proteasome (prosome, macropain) 26S subunit, ATPase, 6	0.00183248	281.257.942	0.54433714	0.35554552	68	8	6.963.235.294	68	0.86057081	493568	0.11087954
13937	10090.ENSMUSP00000120152	P42227	Stat3	Stat3	signal transducer and activator of transcription 3	0.0072854	279.733.164	0.21945701	0.35748353	52	8	4.623.076.923	52	0.86174372	633556	0.07550905
11123	10090.ENSMUSP00000129767	P49722	Psma2		proteasome (prosome, macropain) subunit, alpha type 2	0.00188517	281.194.409	0.53282182	0.35562585	69	8	6.876.811.594	69	0.86061969	503910	
13171	10090.ENSMUSP00000020608	P63330	Ppp2ca	Ppp2ca	protein phosphatase 2 (formerly 2A), catalytic subunit, alpha isoform	0.01601033	257.814.485	0.27059787	0.3878758	111	8	6.463.963.964	111	0.87860424	3274218	
12916	10090.ENSMUSP00000034204	Q9CQF3	Nudt21	Nudt21	nudix (nucleoside diphosphate linked moiety X)-type motif 21	6.85E-6	32.433.291	0.97704918	0.30832517	61	8	812.295.082	61	0.82743622	1982	0.26897188
11125	10090.ENSMUSP00000030212	Q62420	Sh3gl2		SH3-domain GRB2-like 2	0.00186378	282.719.187	0.55974026	0.35370787	56	7	5.971.428.571	56	0.85944678	385056	0.10138249
11126	10090.ENSMUSP00000021306	O08810	Eftud2		elongation factor Tu GTP binding domain containing 2	0.00375683	321.029.225	0.73004695	0.31149812	72	8	7.168.055.556	72	0.82997752	534768	0.21920659
11127	10090.ENSMUSP00000135967	J3KMQ2	Gm5422		predicted pseudogene 5422	0.00492686	277.382.465	0.5054326	0.36051306	71	7	6.864.788.732	71	0.86355195	1038246	0.1054499
12151	10090.ENSMUSP00000113717	G3X9Z4	Pcf11	Pcf11	cleavage and polyadenylation factor subunit homolog (S. cerevisiae)	6.85E-6	32.433.291	0.97704918	0.30832517	61	8	812.295.082	61	0.82743622	1982	0.26897188
11128	10090.ENSMUSP00000007212	Q8VDM4	Psmd2		proteasome (prosome, macropain) 26S subunit, non-ATPase, 2	0.00492686	277.382.465	0.5054326	0.36051306	71	7	6.864.788.732	71	0.86355195	1038246	0.1054499
11129	10090.ENSMUSP00000021049	P62196	Psmc5		protease (prosome, macropain) 26S subunit, ATPase 5	0.00187496	28.252.859	0.52642796	0.35394648	69	8	6.879.710.145	69	0.85959339	470746	0.11168361
12410	10090.ENSMUSP00000087453	O88569	Hnrnpa2b1	Hnrnpa2b1	heterogeneous nuclear ribonucleoprotein A2/B1	0.00221933	31.797.967	0.93548387	0.31448551	62	8	7.972.580.645	62	0.83232333	233852	0.24232768

12666	10090.ENSMUSP00000041902	P22682	Cbl	Cbl	Casitas B-lineage lymphoma	0.00472283	273.506.989	0.37320574	0.36562137	77	8	6.323.376.623	77	0.86653309	908588	
11130	10090.ENSMUSP00000099404	Q62077	Plcg1		phospholipase C, gamma 1	0.01870028	302.795.426	0.24175084	0.33025598	55	8	4.294.545.455	55	0.84400352	5075970	
11131	10090.ENSMUSP00000064261	Q922S8	Kif2c		kinesin family member 2C	0.0037829	305.527.319	0.4996633	0.32730297	55	8	5.041.818.182	55	0.84190206	589986	
11388	10090.ENSMUSP00000028817	P17918	Pcna	Pcna	proliferating cell nuclear antigen	0.01449611	278.208.386	0.23611111	0.3594428	64	7	62.359.375	64	0.86291663	1133828	
11132	10090.ENSMUSP00000021091	P63005	Pafah1b1		platelet-activating factor acetylhydrolase, isoform 1b, subunit 1	0.00658241	297.141.042	0.45324675	0.33654052	56	8	4.991.071.429	56	0.84835304	806672	
11133	10090.ENSMUSP00000098897	Q9Z1B5	Mad2l1		MAD2 mitotic arrest deficient-like 1	0.00280869	287.102.922	0.51243781	0.34830715	67	8	6.714.925.373	67	0.85607468	464140	
11134	10090.ENSMUSP00000021595	P62192	Psmc1		protease (prosome, macropain) 26S subunit, ATPase 1	0.00113961	282.846.252	0.52898551	0.35354897	69	8	6.826.086.957	69	0.85934904	430908	0.11227117
11135	10090.ENSMUSP00000022256	Q99JI4	Psmd6		proteasome (prosome, macropain) 26S subunit, non-ATPase, 6	9,74E+00	283.418.043	0.53623188	0.35283569	69	8	6.856.521.739	69	0.8589092	408594	0.11351857
11136	10090.ENSMUSP00000087457	P09055	Itgb1		integrin beta 1 (fibronectin receptor beta)	0.00740168	313.087.675	0.25387755	0.31939935	50	8	37.52	50	0.8360864	1325574	0.10617564
11137	10090.ENSMUSP00000024727	Q6A068	Cdc5l		cell division cycle 5-like (S. pombe)	0.00856412	321.346.887	0.71244131	0.31119019	72	8	6.941.666.667	72	0.82973316	1281404	0.22155662
11138	10090.ENSMUSP00000084252	P52432	Polr1c		polymerase (RNA) I polypeptide C	0.00777053	299.047.014	0.39935065	0.33439558	56	8	5.330.357.143	56	0.84688691	602366	0.12542017
11139	10090.ENSMUSP00000102857	O35226	Psmd4		proteasome (prosome, macropain) 26S subunit, non-ATPase, 4	0.00118691	283.354.511	0.52132505	0.3529148	70	8	6.784.285.714	70	0.85895807	473918	0.11232261
11140	10090.ENSMUSP00000095866	Q8BWG8	Arrb1		arrestin, beta 1	0.00597543	279.987.294	0.51242938	0.35715907	60	7	58.55	60	0.86154824	697982	
11141	10090.ENSMUSP00000082053	Q60996	Ppp2r5c		protein phosphatase 2, regulatory subunit B (B56), gamma isoform	0.00369598	268.996.188	0.41835443	0.37175248	80	8	71.825	80	0.87000293	1151220	
11142	10090.ENSMUSP00000035551	P35455	Avp		arginine vasopressin	0.00651945	286.340.534	0.47749854	0.34923452	59	8	5.916.949.153	59	0.85666113	478100	
11143	10090.ENSMUSP00000020203	P62307	Snrpf		small nuclear ribonucleoprotein polypeptide F	6,29E-01	314.930.114	0.73898556	0.31753076	74	8	7.263.513.514	74	0.83466914	234504	0.20403128
11144	10090.ENSMUSP00000128400	P62305	Snrpe		small nuclear ribonucleoprotein E	6,29E-01	314.930.114	0.73898556	0.31753076	74	8	7.263.513.514	74	0.83466914	234504	0.20403128
11145	10090.ENSMUSP00000043123	Q99MR6	Srrt		serrate RNA effector molecule homolog (Arabidopsis)	5,04E+00	313.532.402	0.74126126	0.3189463	75	8	72.24	75	0.83574431	94358	0.21564179
11146	10090.ENSMUSP00000018156	P60764	Rac3		RAS-related C3 botulinum substrate 3	0.00192355	306.226.175	0.23961039	0.32655602	56	8	3.741.071.429	56	0.84136448	223668	
11147	10090.ENSMUSP00000025642	Q99KP6	Prpf19		PRP19/PSO4 pre-mRNA processing factor 19 homolog (S. cerevisiae)	0.00422121	293.837.357	0.69036227	0.34032432	77	8	8.024.675.325	77	0.85089434	741182	
11148	10090.ENSMUSP00000002551	P62315	Snrpd1		small nuclear ribonucleoprotein D1	8,69E+00	31.454.892	0.71927928	0.31791557	75	8	7.170.666.667	75	0.83496237	280594	0.20029795
11149	10090.ENSMUSP00000001202	Q6NVF0	Ocrl		oculocerebrorenal syndrome of Lowe	0.00721005	27.750.953	0.44330776	0.36034799	69	7	6.320.289.855	69	0.86345421	1396300	0.09738505
11150	10090.ENSMUSP00000037597	P62317	Snrpd2		small nuclear ribonucleoprotein D2	7,53E+00	314.866.582	0.72432432	0.31759483	75	8	71.88	75	0.83471801	297690	0.20191011
11151	10090.ENSMUSP00000019362	Q60838	Dvl2		dishevelled 2, dsh homolog (Drosophila)	0.0117699	274.523.507	0.34630631	0.36426753	75	8	6.085.333.333	75	0.86575115	922190	0.08817391
11152	10090.ENSMUSP00000105709	Q61380	Calm1		calmodulin 1	0.01944196	287.992.376	0.0961039	0.34723141	56	8	3.289.285.714	56	0.85539048	1417878	
11153	10090.ENSMUSP00000099488	P27048	Snrpb		small nuclear ribonucleoprotein B	8,93E+00	314.485.388	0.70631579	0.3179798	76	8	71.0	76	0.83501124	305796	0.19832402
11410	10090.ENSMUSP00000023462	P63085	Mapk1	Mapk1	mitogen-activated protein kinase 1	0.02438013	27.064.803	0.12684145	0.36948357	90	8	3.918.888.889	90	0.86873228	3462724	
11154	10090.ENSMUSP00000030404	Q9QZH3	Ppie		peptidylprolyl isomerase E (cyclophilin E)	0.01420515	285.387.548	0.63386076	0.35040071	80	7	78.425	80	0.85739419	1261056	
13459	10090.ENSMUSP00000029270	P51943	Ccna2	Ccna2	cyclin A2	0.00323864	289.771.283	0.40092166	0.34509976	63	8	5.988.888.889	63	0.85402209	447402	
13971	10090.ENSMUSP00000068530	Q6PFD9	Nup98	Nup98	nucleoporin 98	0.00212911	301.143.583	0.52308591	0.33206751	59	8	6.430.508.475	59	0.84527417	539728	0.1509509
11155	10090.ENSMUSP00000036384	Q05144	Rac2		RAS-related C3 botulinum substrate 2	0.00222073	305.146.125	0.24016939	0.32771185	58	8	3.767.241.379	58	0.84219529	246790	
11412	10090.ENSMUSP00000015892	Q922U1	Prpf3	Prpf3	PRP3 pre-mRNA processing factor 3 homolog (yeast)	0.00320772	323.379.924	0.8960452	0.30923379	60	8	7.748.333.333	60	0.82816929	390900	0.25151515
11156	10090.ENSMUSP00000024739	P11499	Hsp90ab1		heat shock protein 90 alpha (cytosolic), class B member 1	0.01383563	269.440.915	0.11373261	0.37113888	58	7	3.586.206.897	58	0.86966083	741282	0.04994469
13973	10090.ENSMUSP00000066789	B2RWS6	Ep300	Ep300	E1A binding protein p300	0.02885933	261.817.027	0.22727273	0.38194613	89	7	53.0	89	0.87552536	2513792	0.06743003
11157	10090.ENSMUSP00000034742	P30276	Ccnb2		cyclin B2	0.00161142	311.181.703	0.40225989	0.32135566	60	8	4.833.333.333	60	0.83755254	287020	0.12923351
11414	10090.ENSMUSP00000107576	Q91Y86	Mapk8	Mapk8	mitogen-activated protein kinase 8	0.0125412	275.285.896	0.10168651	0.36325871	64	8	35.859.375	64	0.8651647	893882	
12694	10090.ENSMUSP00000023507	Q9WV60	Gsk3b	Gsk3b	glycogen synthase kinase 3 beta	0.02862611	255.717.916	0.21880342	0.3910559	91	7	6.527.472.527	91	0.88021699	3658502	
11158	10090.ENSMUSP00000059501	O70404	Vamp8		vesicle-associated membrane protein 8	0.00415	28.703.939	0.52406134	0.34838424	62	7	5.493.548.387	62	0.85612355	720590	0.09792421
11159	10090.ENSMUSP00000075614	Q3ULL6	Upf3b		UPF3 regulator of nonsense transcripts homolog B (yeast)	0.00126633	291.359.593	0.65648148	0.34321849	81	8	7.749.382.716	81	0.85280031	415488	0.14141209
11672	10090.ENSMUSP00000111457	Q9D2G5	Synj2	Synj2	synaptojanin 2	0.00241751	287.801.779	0.61959184	0.34746137	50	8	71.94	50	0.85553709	700058	0.1242487
12952	10090.ENSMUSP00000096549	P70372	Elavl1	Elavl1	ELAV (embryonic lethal, abnormal vision)-like 1 (Hu antigen R)	0.0056937	305.972.046	0.80054274	0.32682724	67	7	7.653.731.343	67	0.84155996	478074	
11160	10090.ENSMUSP00000020397	P62320	Snrpd3		small nuclear ribonucleoprotein D3	0.00135528	314.358.323	0.67266067	0.31810833	78	8	6.926.923.077	78	0.83510898	398212	0.19348947
11161	10090.ENSMUSP00000100599	A0A0N4SUH6	ENSMUSG00000078184		RIKEN cDNA B020018G12 gene	0.00133476	291.232.529	0.64378199	0.34336824	82	8	7.682.926.829	82	0.85289805	427026	0.13994402
11162	10090.ENSMUSP00000034560	Q7TNP2	Ppp2r1b		protein phosphatase 2 (formerly 2A), regulatory subunit A (PR 65), beta isoform	0.00496167	26.639.136	0.37400821	0.37538755	86	8	683.255.814	86	0.87200665	1398022	0.09158925
11419	10090.ENSMUSP00000023829	P39689	Cdkn1a	Cdkn1a	cyclin-dependent kinase inhibitor 1A (P21)	0.0038623	278.462.516	0.41885522	0.35911476	55	8	6.825.454.545	55	0.86272114	402266	0.10323202
11163	10090.ENSMUSP00000058757	P35235	Ptpn11		protein tyrosine phosphatase, non-receptor type 11	0.00598659	281.448.539	0.33333333	0.35530474	66	8	6.422.727.273	66	0.8604242	1043664	
11164	10090.ENSMUSP00000030348	P61327	Magoh		mago-nashi homolog, proliferation-associated (Drosophila)	0.00133476	291.232.529	0.64378199	0.34336824	82	8	7.682.926.829	82	0.85289805	427026	0.13994402
11165	10090.ENSMUSP00000052262	P70280	Vamp7		vesicle-associated membrane protein 7	0.00356242	287.611.182	0.52022529	0.34769163	63	7	5.479.365.079	63	0.85568371	719486	0.09802084
11166	10090.ENSMUSP00000021283	Q5SW28	Pik3r5		phosphoinositide-3-kinase, regulatory subunit 5, p101	0.00226015	293.074.968	0.32670807	0.34120962	70	8	4.852.857.143	70	0.85148079	576626	0.10216541
12703	10090.ENSMUSP00000014911	P29037	Tbp	Tbp	TATA box binding protein	0.00538337	294.917.408	0.4039548	0.33907798	60	8	53.6	60	0.85006353	692166	0.11308017
11167	10090.ENSMUSP00000085867	Q99M28	Rnps1		ribonucleic acid binding protein S1	0.00260091	290.914.867	0.64119894	0.34374318	83	8	763.373.494	83	0.85314241	515008	
13984	10090.ENSMUSP00000044548	Q9CWZ3	Rbm8a	Rbm8a	RNA binding motif protein 8a	0.00133476	291.232.529	0.64378199	0.34336824	82	8	7.682.926.829	82	0.85289805	427026	0.13994402
11168	10090.ENSMUSP00000062864	Q9JHG7	Pik3cg		phosphoinositide-3-kinase, catalytic, gamma polypeptide	0.00168898	292.757.306	0.34464043	0.34157986	67	8	5.043.283.582	67	0.85172515	460324	
11169	10090.ENSMUSP00000023165	P45481	Crebbp		CREB binding protein	0.01388103	279.606.099	0.25282678	0.35764599	67	8	4.840.298.507	67	0.86184146	1287640	0.08000493
11170	10090.ENSMUSP00000101315	O35904	Pik3cd		phosphatidylinositol 3-kinase catalytic delta polypeptide	0.00164596	292.566.709	0.35123615	0.34180239	69	8	5.036.231.884	69	0.85187176	457642	
13475	10090.ENSMUSP00000091495	P25322	Ccnd1	Ccnd1	cyclin D1	0.00896271	278.526.048	0.33710407	0.35903285	52	8	6.409.615.385	52	0.86267227	640920	
11171	10090.ENSMUSP00000105663	P34152	Ptk2		PTK2 protein tyrosine kinase 2	0.01130507	292.884.371	0.27536232	0.34143167	70	8	4.922.857.143	70	0.85162741	2451752	0.09690664
11172	10090.ENSMUSP00000009774	P62715	Ppp2cb		protein phosphatase 2 (formerly 2A), catalytic subunit, beta isoform	0.01009114	265.628.971	0.34236874	0.37646496	91	8	6.686.813.187	91	0.8725931	1804654	
11173	10090.ENSMUSP00000007130	Q02248	Ctnnb1		catenin (cadherin associated protein), beta 1	0.02386475	263.024.142	0.22215692	0.38019324	83	7	6.107.228.916	83	0.87459681	2077324	0.07750953
11174	10090.ENSMUSP00000006565	Q9JJ66	Cdc20		cell division cycle 20	0.00426761	281.702.668	0.40905084	0.35498421	83	8	6.178.313.253	83	0.86022872	785764	
11175	10090.ENSMUSP00000070728	Q3ULA2	Btrc		beta-transducin repeat containing protein	0.00491211	291.550.191	0.42721519	0.34299412	80	8	59.75	80	0.8526537	593506	0.11534749
12199	10090.ENSMUSP00000088837	P15208	Insr	Insr	insulin receptor	0.00582887	287.420.584	0.30745098	0.34792219	51	7	5.803.921.569	51	0.85583032	773294	0.11161388
11176	10090.ENSMUSP00000071989	P24860	Ccnb1		cyclin B1	0.00403887	288.945.362	0.35211268	0.34608619	72	8	5.665.277.778	72	0.85465741	706252	0.10171055
13993	10090.ENSMUSP00000051544	O70480	Vamp4	Vamp4	vesicle-associated membrane protein 4	0.00634589	290.914.867	0.52994555	0.34374318	58	8	5.653.448.276	58	0.85314241	880600	0.1020478
11177	10090.ENSMUSP00000086312	Q8R0A0	Gtf2f2		general transcription factor IIF, polypeptide 2	8,39E+00	310.228.717	0.58146067	0.32234282	89	8	6.482.022.472	89	0.8382856	211682	0.18005618
11178	10090.ENSMUSP00000133154	E9PV04	Gm8994		predicted gene 8994	0.00597221	289.898.348	0.54603175	0.3449485	91	8	7.079.120.879	91	0.85392435	1073974	
13995	10090.ENSMUSP00000030797	P63024	Vamp3	Vamp3	vesicle-associated membrane protein 3	0.00493318	287.865.311	0.55584416	0.34738468	56	7	5.794.642.857	56	0.85548822	720098	0.10403309
11179	10090.ENSMUSP00000021273	P63044	Vamp2		vesicle-associated membrane protein 2	0.01025809	278.970.775	0.40677321	0.35846049	73	7	5.093.150.685	73	0.86233017	1102946	0.078598
11180	10090.ENSMUSP00000034296	O08908	Pik3r2		phosphatidylinositol 3-kinase, regulatory subunit, polypeptide 2 (p85 beta)	0.00314944	290.533.672	0.31442242	0.34419418	77	8	4.812.987.013	77	0.85343564	717648	0.0988293
13228	10090.ENSMUSP00000020099	P11440	Cdk1	Champ1	cyclin-dependent kinase 1	0.01424783	270.139.771	0.24511082	0.37017874	118	8	5.008.474.576	118	0.86912325	1643918	0.07175465
11181	10090.ENSMUSP00000004990	Q9QZ80	Mapk14		mitogen-activated protein kinase 14	0.02259679	274.015.248	0.13584475	0.36494319	73	8	3.591.780.822	73	0.86614212	1423324	
11182	10090.ENSMUSP00000026667	Q91VC3	Eif4a3		eukaryotic translation initiation factor 4A3	0.00597221	289.898.348	0.54603175	0.3449485	91	8	7.079.120.879	91	0.85392435	1073974	
13999	10090.ENSMUSP00000030642	Q9R1P3	Psmb2	Psmb2	proteasome (prosome, macropain) subunit, beta type 2	0.00489816	277.128.335	0.52387042	0.36084365	69	7	6.997.101.449	69	0.86374743	1002188	
11183	10090.ENSMUSP00000031583	E9Q4Z2	Acacb		acetyl-Coenzyme A carboxylase beta	0.03031524	29.523.507	0.06628622	0.33871315	68	7	2.029.411.765	68	0.84981918	3322922	
11184	10090.ENSMUSP00000002733	Q3THK3	Gtf2f1		general transcription factor IIF, polypeptide 1	0.00142969	309.339.263	0.54825609	0.32326967	92	8	6.333.695.652	92	0.8389698	260862	0.17164487
11185	10090.ENSMUSP00000020843	Q705X9	Acaca		acetyl-Coenzyme A carboxylase alpha	0.03170336	296.886.912	0.06606991	0.33682859	69	7	1.963.768.116	69	0.84854853	3438912	
12209	10090.ENSMUSP00000000137	P61161	Actr2	Actr2	ARP2 actin-related protein 2	0.00332206	279.606.099	0.56240929	0.35764599	53	8	6.390.566.038	53	0.86184146	276610	
11186	10090.ENSMUSP00000031697	Q9WTX6	Cul1		cullin 1	0.00387858	292.503.177	0.40336808	0.34187663	87	8	5.854.022.989	87	0.85192063	730568	
11187	10090.ENSMUSP00000038744	Q9WTX5	Skp1a		S-phase kinase-associated protein 1A	0.00674441	287.102.922	0.38815117	0.34830715	89	8	5.868.539.326	89	0.85607468	859696	0.10608123
11188	10090.ENSMUSP00000045487	Q9CR99	ENSMUSG00000036463		RIKEN cDNA 4930544G11 gene	0.01495758	270.330.368	0.19845679	0.36991774	81	8	4.860.493.827	81	0.86897664	1640570	0.06722675
11189	10090.ENSMUSP00000033154	Q07832	Plk1		polo-like kinase 1	0.01025227	270.775.095	0.32258772	0.36931018	96	8	5.752.083.333	96	0.86863454	1173074	
11190	10090.ENSMUSP00000029653	P01132	Egf		epidermal growth factor	0.01532933	26.111.817	0.2912377	0.38296837	94	8	5.942.553.191	94	0.87606295	1944182	0.07481644
11191	10090.ENSMUSP00000026572	Q61411	Hras1		Harvey rat sarcoma virus oncogene 1	0.00993141	265.057.179	0.23071161	0.37727709	90	8	5.701.111.111	90	0.87303294	1663234	
11192	10090.ENSMUSP00000021698	P07901	Hsp90aa1		heat shock protein 90, alpha (cytosolic), class A member 1	0.0321659	251.397.713	0.13089133	0.39777609	91	7	4.595.604.396	91	0.88354022	2230398	0.05083633
11193	10090.ENSMUSP00000023460	Q9CQ49	Ncbp2		nuclear cap binding protein subunit 2	0.00417366	285.387.548	0.48151606	0.35040071	104	7	6.696.153.846	104	0.85739419	869294	0.11686132
11194	10090.ENSMUSP00000051619	Q63844	Mapk3		mitogen-activated protein kinase 3	0.02114768	270.711.563	0.12844933	0.36939686	91	8	3.821.978.022	91	0.86868341	3069558	

11195	10090.ENSMUSP00000043204	P62876	Polr2l		polymerase (RNA) II (DNA directed) polypeptide L	0.00512912	27.566.709	0.44669436	0.3627564	108	7	6.709.259.259	108	0.86487147	1249634	0.10353795
11196	10090.ENSMUSP00000023036	P62878	Rbx1		ring-box 1	0.01142002	272.808.132	0.28680397	0.36655799	115	7	6.122.608.696	115	0.86707067	1559886	0.08884962
11197	10090.ENSMUSP00000007708	Q76MZ3	Ppp2r1a		protein phosphatase 2 (formerly 2A), regulatory subunit A (PR 65), alpha isoform	0.02307964	252.668.361	0.23147107	0.39577571	127	8	6.048.818.898	127	0.8825628	3844122	
12221	10090.ENSMUSP00000094225	Q9Z0Z3	Skp2	Skp2	S-phase kinase-associated protein 2 (p45)	0.00144771	305.463.787	0.55133424	0.32737105	67	8	6.155.223.881	67	0.84195093	246860	0.14551357
12477	10090.ENSMUSP00000030464	Q64143	Pik3r3	Pik3r3	phosphatidylinositol 3 kinase, regulatory subunit, polypeptide 3 (p55)	0.00215261	29.364.676	0.33695161	0.34054522	67	8	488.358.209	67	0.85104095	565200	0.10412755
11198	10090.ENSMUSP00000079380	P63001	Rac1		RAS-related C3 botulinum substrate 1	0.01704978	275.540.025	0.16404582	0.36292368	102	8	3.787.254.902	102	0.86496921	1292500	
11199	10090.ENSMUSP00000001780	P31750	Akt1		thymoma viral proto-oncogene 1	0.03418171	262.198.221	0.13962264	0.38139084	106	7	4.398.113.208	106	0.87523214	2679608	
12479	10090.ENSMUSP00000056774	P26450	Pik3r1	Pik3r1	phosphatidylinositol 3-kinase, regulatory subunit, polypeptide 1 (p85 alpha)	0.00649973	283.989.835	0.24036281	0.35212528	99	8	4.326.262.626	99	0.85846936	1068604	0.082405
11200	10090.ENSMUSP00000019882	P60898	Polr2i		polymerase (RNA) II (DNA directed) polypeptide I	0.00515223	279.606.099	0.40179462	0.35764599	119	7	642.605.042	119	0.86184146	1364972	0.10534509
11968	10090.ENSMUSP00000005164	P68181	Prkacb	Prkacb	protein kinase, cAMP dependent, catalytic, beta	0.01162591	284.371.029	0.12039743	0.35165326	59	8	3.069.491.525	59	0.85817613	1086400	
11201	10090.ENSMUSP00000021090	Q60631	Grb2		growth factor receptor bound protein 2	0.01486067	257.560.356	0.25954962	0.38825851	110	8	5.608.181.818	110	0.87879973	2528764	
11969	10090.ENSMUSP00000005606	P05132	Prkaca	Prkaca	protein kinase, cAMP dependent, catalytic, alpha	0.01716131	274.904.701	0.1347649	0.36376242	74	8	3.371.621.622	74	0.86545792	1528522	
11202	10090.ENSMUSP00000093980	P62488	Polr2g		polymerase (RNA) II (DNA directed) polypeptide G	0.00526422	279.034.307	0.39733894	0.35837887	120	7	6.398.333.333	120	0.8622813	1392122	0.10386905
11203	10090.ENSMUSP00000015800	Q3U9G0	Hspa8		heat shock protein 8	0.0412094	260.038.119	0.31771918	0.384559	132	7	6.366.666.667	132	0.87689375	3700956	
11204	10090.ENSMUSP00000031167	Q8CFI7	Polr2b		polymerase (RNA) II (DNA directed) polypeptide B	0.00583807	278.398.983	0.38707492	0.35919671	122	7	6.343.442.623	122	0.86277001	1450598	0.10182091
12996	10090.ENSMUSP00000044305	Q9DC48	Cdc40	Cdc40	cell division cycle 40	0.00132521	323.443.456	0.82813207	0.30917305	67	8	7.474.626.866	67	0.82812042	449788	0.24506973
11205	10090.ENSMUSP00000051968	Q63871	Polr2k		polymerase (RNA) II (DNA directed) polypeptide K	0.00760569	271.791.614	0.37380645	0.36792894	125	7	62.768	125	0.8678526	1707854	0.09285207
11206	10090.ENSMUSP00000104298	P02340	Trp53		transformation related protein 53	0.06186778	253.875.476	0.1137931	0.39389389	116	8	4.556.034.483	116	0.88163425	5271798	0.05312399
11718	10090.ENSMUSP00000020329	Q01279	Egfr	Egfr	epidermal growth factor receptor	0.02240689	252.604.828	0.23032714	0.39587525	117	8	5.768.376.068	117	0.88261167	3612248	
11207	10090.ENSMUSP00000021405	Q923G2	Polr2h		polymerase (RNA) II (DNA directed) polypeptide H	0.00846493	269.949.174	0.36085138	0.3704401	128	7	6.209.375	128	0.86926987	2007212	0.08999094
11208	10090.ENSMUSP00000090237	P05480	Src		Rous sarcoma oncogene	0.02501912	264.485.388	0.19726324	0.37809272	122	7	4.726.229.508	122	0.87347278	2770128	0.06526809
11209	10090.ENSMUSP00000004786	Q80UW8	Polr2e		polymerase (RNA) II (DNA directed) polypeptide E	0.00858668	269.885.642	0.35719477	0.37052731	129	7	6.180.620.155	129	0.86931874	2032908	0.08957421
11210	10090.ENSMUSP00000043566	P61219	Polr2f		polymerase (RNA) II (DNA directed) polypeptide F	0.01795603	266.581.957	0.31418597	0.37511916	138	7	5.835.507.246	138	0.87186003	2469596	0.08104871
11211	10090.ENSMUSP00000080543	D3YYZ2	Gm5239		predicted pseudogene 5239	0.07577783	236.340.534	0.10805452	0.42311828	270	7	4.564.814.815	270	0.89512267	10281420	

Supplementary Table S5. A focus on PPI network hubs

SUID	Database Identifier	Canonical Name	Name	Description	Average Shortest Path Length	Betweenness Centrality	Closeness Centrality	Clustering Coefficient	Degree	Eccentricity	Neighborhood Connectivity	Number Of Undirected Edges	Query Term	Radiality	Topological Coefficient
11212	10090.ENSMUSP00000136791	J3QK04	Gm7808	predicted pseudogene 7808	236.340.534	0.07577783	0.42311828	0.10805452	270	7	4.564.814.815	270		0.89512267	0.04686668
12018	10090.ENSMUSP00000114180	Q9ET24	Ubc	ubiquitin C	240.025.413	0.05482875	0.41662255	0.12827787	235	7	4.778.297.872	235	Ubc	0.89228814	0.04982584
11206	10090.ENSMUSP00000104298	P02340	Trp53	transformation related protein 53	253.875.476	0.06186778	0.39389389	0.1137931	116	8	4.556.034.483	116		0.88163425	0.05312399
11211	10090.ENSMUSP00000080543	D3YYZ2	Gm5239	predicted pseudogene 5239	236.340.534	0.07577783	0.42311828	0.10805452	270	7	4.564.814.815	270		0.89512267	

Supplementary Table S6. DEGs deregulated in *Cx30*$^{-/-}$ sorted in ascending order of fold change (FC).

Probe name, gene symbol, description and FC are shown in this table for each one of the 57 out 81 DEGs that encode 56 proteins.

ProbeName	GeneSymbol	Description	FC
A_55_P2934160	*Gjb6*	Mus musculus gap junction protein – Transcript variant 3	-85.6744
A_52_P482251	*Gjb6*	Mus musculus gap junction protein – Transcript variant 2	-5.46416
A_51_P502906	*Ang4*	Angiogenin, ribonuclease A family, member 4(Ang4)	-4.51398
A_52_P560996	*Gm26782*	Mus musculus adult male colon cDNA	-4.25546
A_52_P98778	*Ang4*	Angiogenin, ribonuclease A family, member 4(Ang4)	-3.39696
A_52_P91019	*Olfr1386*	Mus musculus olfactory receptor 1386 (Olfr1386)	-2.84102
A_52_P382886	*Gjb2*	Mus musculus gap junction protein	-2.81565
A_55_P2030667	*Eda*	Mus musculus ectodysplasin-A (Eda)	-2.77284
A_51_P338031	*Trpm1*	Mus musculus transient receptor potential cation channel	-2.68726
A_51_P230537	*Ccdc114*	Mus musculus coiled-coil domain containing 114 (Ccdc114)	-2.51915
A_52_P277082	*Gpr143*	Mus musculus G protein-coupled receptor 143 (Gpr143)	-2.43662
A_52_P680761	*Tdrd6*	Mus musculus tudor domain containing 6 (Tdrd6)	-2.34165
A_51_P485985	*Mbnl3*	Mus musculus muscleblind-like 3 (Drosophila) (Mbnl3)	-2.31374
A_51_P144024	*Trpa1*	Mus musculus transient receptor potential cation channel	-2.29293
A_65_P11062	*Gm36463*	PREDICTED: Mus musculus predicted gene	-2.27591
A_55_P2173952	*Myh6*	Mus musculus myosin	-2.24038
A_55_P2716656	*Pgd*	phosphogluconate dehydrogenase [Source:MGI Symbol]	-2.23892
A_55_P2924285	*Dusp14*	dual specificity phosphatase 14 [Source:MGI Symbol]	-2.21072
A_66_P137343	*Enpp6*	Mus musculus 0 day neonate head cDNA	-2.21023
A_52_P208416	*Olfr1431*	Mus musculus olfactory receptor 1431 (Olfr1431)	-2.19804
A_66_P105262	*Hspb11*	PREDICTED: Mus musculus heat shock protein family B (small)	-2.07118
A_55_P2714597	*Bhmt*	Mus musculus betaine-homocysteine methyltransferase (Bhmt)	-2.05747
A_55_P2483194	*Wfdc18*	Mus musculus WAP four-disulfide core domain 18 (Wfdc18)	-2.04821
A_52_P556462	*Fancd2*	Mus musculus Fanconi anemia	-2.03914
A_55_P2824451	*Fam167a*	Mus musculus family with sequence similarity 167	-2.00453
A_51_P201751	*Olfr870*	Mus musculus olfactory receptor 870 (Olfr870)	-1.89078
A_51_P100625	*Apon*	Mus musculus apolipoprotein N (Apon)	-1.88786
A_55_P2716491	*Ang6*	Mus musculus angiogenin	-4.3259
A_51_P391159	*Ang*	angiogenin, ribonuclease, RNase A family, 5	-3.3733
A_66_P132845	*Prg3*	Mus musculus proteoglycan 3 (Prg3)	-3.1445
A_52_P25420	*Srcin1*	Mus musculus SRC kinase signaling inhibitor 1 (Srcin1)	-2.4185

A_55_P2180949	*Lcp2*	lymphocyte cytosolic protein 2 [Source:MGI Symbol	-2.2661
A_55_P2737912	*Lgi3*	Mus musculus 10 days neonate medulla oblongata cDNA	-2.1291
A_55_P2743344	*Svil*	supervillin [Source:MGI Symbol]	1.73276
A_51_P321610	*Mapkbp1*	Mus musculus mRNA for JNK-binding protein JNKBP1	2.36026
A_66_P103780	*Gm3646*	Mus musculus predicted gene 3646 (Gm3646)	2.80451
A_55_P2735845	*Tnfrsf10b*	tumor necrosis factor receptor superfamily, member 10b [Source:MGI Symbol]	3.05238
A_55_P2069765	*Kiss1*	Mus musculus KiSS-1 metastasis-suppressor (Kiss1)	1.678236
A_51_P309854	*Kcnn2*	Mus musculus potassium intermediate/small conductance calcium-activated channel	1.688343
A_52_P127572	*Elf4*	Mus musculus E74-like factor 4 (ets domain transcription factor) (Elf4)	1.731594
A_55_P2720113	*Pamr1*	peptidase domain containing associated with muscle regeneration 1 [Source:MGI Symbol]	1.874715
A_66_P110610	*Clcnkb*	Mus musculus chloride channel Kb (Clcnkb)	2.040674
A_55_P2740248	*Pkd1*	Mus musculus cDNA	2.050697
A_55_P2208300	*Zfp950*	PREDICTED: Mus musculus RIKEN cDNA 5830428H23 gene (5830428H23Rik)	2.078884
A_55_P2715301	*Reln*	reelin [Source:MGI Symbol	2.167367
A_55_P1984881	*Fyb2*	FYN binding protein 2	2.385044
A_52_P160418	*Ubr3*	Mus musculus ubiquitin protein ligase E3 component n-recognin 3 (Ubr3)	2.454792
A_55_P2783542	*Rasgrf2*	Mus musculus RAS protein-specific guanine nucleotide-releasing factor 2 (Rasgrf2)	2.465768
A_55_P2010811	*Olfr59*	Mus musculus olfactory receptor 59 (Olfr59)	2.492033
A_51_P134923	*Rnf148*	Mus musculus ring finger protein 148 (Rnf148)	2.560107
A_55_P2804650	*Il1r2*	Mus musculus interleukin 1 receptor	2.731522
A_52_P161411	*Usp4*	ubiquitin specific peptidase 4 (proto-oncogene) [Source:MGI Symbol]	2.751463
A_55_P1996893	*Zpbp2*	Mus musculus zona pellucida binding protein 2 (Zpbp2)	2.767583
A_55_P2040873	*Gm867*	Mus musculus predicted gene 867 (Gm867)	2.901609
A_66_P130275	*Gm4491*	PREDICTED: Mus musculus predicted gene 4491 (Gm4491)	2.918621
A_52_P565847	*AU018091*	Mus musculus expressed sequence AU018091 (AU018091)	3.147764
A_51_P464703	*Ccl8*	Mus musculus chemokine (C-C motif) ligand 8 (Ccl8)	3.506606
A_55_P2737692	*Npy1r*	Mus musculus neuropeptide Y receptor Y1 (Npy1r)	3.697697

Supplementary Table S7. DEGs belonging to the pair comparison Cx30wt vs Cx30KO. Information about feature type, description and regulation are shown in the table for each of the 15 out 81 DEGs that encode different types of non-coding RNAs.

Feature Type	Description	Regulation
lincRNA	lincRNA:chr4:98943050-98959998 forward strand	up
lincRNA	lincRNA:chr1:175660642-175671878 reverse strand	up
lincRNA	lincRNA:chr6:131263850-131314850 reverse strand	up
lincRNA	lincRNA:chr4:53312709-53355184 forward strand	down
lincRNA	lincRNA:chr15:58327703-58329443 reverse strand	down
lincRNA	lincRNA:chr6:31037606-31037912 reverse strand	up
lincRNA	lincRNA:chr2:104590593-104606218 forward strand	up
lincRNA	lincRNA:chrX:133315875-133338600 reverse strand	up
ncRNA	A930018M24Rik - Mus musculus adult male diencephalon cDNA	up
antisense lncRNA	B230369F24Rik - Mus musculus adult pancreas islet cells cDNA	up
antisense lncRNA	9530036O11Rik - Mus musculus RIKEN cDNA 9530036O11Rik	up
lncRNA	Gm33951 - PREDICTED: Mus musculus predicted gene	down
ncRNA	Gm14169 - Mus musculus predicted gene 14169	up
ncRNA	Gm13429 - Mus musculus 0 day neonate lung cDNA	up
lncRNA	Gm20098 - Mus musculus predicted gene	down

Supplementary Table S8. Correlation between deregulated miRNAs and predicted/ validated deregulated miRNA targets. The table shows that only one pair of miRNA/mRNA has an inverse correlation between expression levels (up vs down), i.e. mmu-miR-29b-3p and *Gjb2*.

Cx30^{wt} vs Cx30^{ko}

Deregulated miRNAs	Regulation	DEGs	Regulation
mmu-miR-29b-3p	up	*Elf4*	Up
		Pkd1	Up
		AU018091	Up
mmu-miR-34c-5p	up	*Gjb2*	Down
		Elf4	Up
mmu-miR-335-5p	up	*Elf4*	Up
mmu-miR-181a-1-3p	up	*Reln*	Up
